# Structure–Function Relationships of a High-Molecular-Weight Exopolysaccharide from *Euhalothece* sp. with Bioactive and Emulsifying Properties

**DOI:** 10.1021/acsomega.6c04930

**Published:** 2026-10-26

**Authors:** Kübra Şentürk Azak, Mark Klöpfer, Emel Tamahkar Irmak, Gökçe Taner, Wolfgang Eisenreich, Mete Yilmaz

**Affiliations:** † Department of Bioengineering, 226838 Bursa Technical University, 16310 Bursa, Turkey; ‡ Bavarian NMR Center, Structural Membrane Biochemistry, Department of Chemistry, 9184 Technical University of Munich, Lichtenbergstr. 4, 85747 Garching, Germany

## Abstract

Cyanobacterial exopolysaccharides (EPSs) are promising sustainable biopolymers, yet halotolerant strains remain comparatively underexplored and the relationships between EPS composition and functionality are still not well-defined. In this study, an EPS produced by *Euhalothece* sp. AQUAMEB-3 was isolated and characterized to assess its physicochemical properties, compositional features, and multifunctional performance. The EPS was identified as a high-molecular-weight (*M*_w_ ∼ 2617 kDa), strongly anionic heteropolysaccharide with a zeta potential of −45.1 mV, composed mainly of xylose, fucose, amino sugars, and uronic acids. FTIR, ^1^H NMR, ^1^H–^1^H correlation spectroscopy (COSY) NMR, and GC–MS analyses indicated a structurally heterogeneous polymer, while XRD and SEM revealed a predominantly amorphous and porous morphology. The EPS also exhibited pronounced shear-thinning behavior and strong water affinity. Functionally, the EPS showed high emulsifying performance, with *E*_24_ values reaching 94.2% at 1.0% (w/v), comparable to xanthan gum under the tested conditions, and maintained emulsion stability over extended storage. It also displayed antibacterial activity against *Escherichia coli* (MIC: 150 μg mL^–1^) and *Staphylococcus aureus* (MIC: 300 μg mL^–1^), together mL^–1^ with concentration-dependent antioxidant and iron-chelating activities, reaching 82.6% radical scavenging and 96% metal chelation at the highest tested concentrations. Cytotoxicity assays indicated concentration- and cell-type-dependent cytocompatibility. Taken together, these results define a distinct physicochemical and functional profile for the EPS produced by *Euhalothece* sp. AQUAMEB-3 and expand the available characterization data for halotolerant cyanobacterial exopolysaccharides. The observed associations between its compositional and physicochemical characteristics and functional properties should be regarded as correlative rather than mechanistically established. These findings support the potential of AQUAMEB-3 EPS for further investigation in emulsion stabilization and other biobased formulation applications.

## Graphical abstract

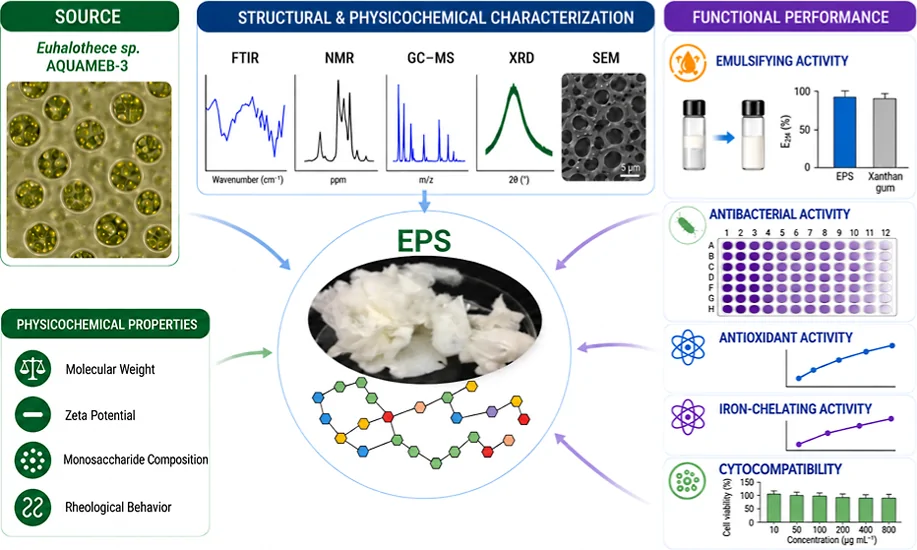

## Introduction

Natural polymers have attracted increasing attention as sustainable alternatives to synthetic materials due to their biodegradability, structural diversity, and functional versatility.^1,2^ Among these, microbial exopolysaccharides (EPSs) represent a particularly promising class of biopolymers, exhibiting a wide range of physicochemical properties that can be tailored by their monosaccharide composition, molecular weight, and functional group distribution.^3,4^

Cyanobacterial EPSs are high-molecular-weight heteropolymers composed of various monosaccharides, including hexoses, pentoses, deoxy sugars, and uronic acids, often substituted with sulfate or phosphate groups.^5,6^ This structural complexity results in polymers with unique physicochemical characteristics such as high viscosity, water-binding capacity, and polyelectrolyte behavior.^7^ In particular, the presence of uronic acids and charged substituents imparts strong anionic character, which governs intermolecular interactions, solution behavior, and functional properties such as emulsification, metal ion binding, and interactions with biological systems.^8,9^

The macroscopic performance of polysaccharide-based polymers is closely governed by their structure–property relationships.^10^ Parameters such as molecular weight, chain flexibility, and degree of branching affect rheological behavior, thermal stability, and phase interactions. High-molecular-weight polysaccharides typically exhibit shear-thinning behavior due to polymer chain entanglement and alignment under applied shear, making them suitable for applications as thickeners, stabilizers, and injectable materials.^11^ Similarly, amphiphilic polysaccharides containing both hydrophilic and hydrophobic moieties can act as effective bioemulsifiers by stabilizing oil–water interfaces.^12^

In addition to their physicochemical functionality, increasing attention has been directed toward the biological activities of microbial EPSs, including antibacterial, antioxidant, and metal-chelating properties.^13,14^ These bioactivities are closely associated with the presence of functional groups such as hydroxyl, carboxyl, and amino moieties, as well as the overall charge distribution and molecular architecture of the polymer.^15^ Such combined physicochemical and biological functionalities are particularly desirable for the development of advanced bioactive materials.^16^ These multifunctional properties are particularly relevant for biomaterial applications requiring simultaneous moisture retention, antimicrobial activity, and interfacial stability, such as wound-associated systems.^17,18^

Despite the growing interest in microbial polysaccharides, cyanobacterial EPSs remain relatively underexplored in polymer science, particularly those derived from halotolerant strains. While previous studies have primarily focused on glucose-rich or structurally simpler systems, the relationship between compositional heterogeneity and functional performance is still not well established.^3,19^ In particular, systematic correlations between monosaccharide composition and combined physicochemical and biological properties remain limited.^20^ Moreover, EPSs produced by halotolerant cyanobacteria, which may possess distinct structural features arising from environmental adaptation, have not been sufficiently investigated. The role of complex and less common monosaccharides, such as pentoses, deoxy sugars, and amino sugars, in governing interfacial behavior and bioactivity also remains poorly understood.^21,22^

Among cyanobacteria, halotolerant species represent particularly attractive sources of EPS because adaptation to saline environments may promote the production of polymers with enhanced hydration capacity, ionic interactions, and osmotic stability.^23,24^ However, despite its ecological adaptation to hypersaline environments, the physicochemical and functional properties of EPS produced by *Euhalothece* species remain largely unexplored. *Euhalothece* sp. AQUAMEB-3 was selected because it was isolated from a saline habitat and demonstrated the capacity to produce recoverable EPS under laboratory cultivation. The present study therefore addresses this knowledge gap through an integrated evaluation of the molecular weight, charge characteristics, biochemical composition, monosaccharide profile, rheological behavior, and selected functional properties of AQUAMEB-3 EPS. This approach identifies associations between the physicochemical characteristics of EPS from an underexplored halotolerant cyanobacterium and its potential biotechnological functions.

In this study, we report the isolation and characterization of an exopolysaccharide produced by *Euhalothece* sp. AQUAMEB-3. The EPS is identified as a high-molecular-weight (∼2617 kDa) anionic heteropolysaccharide with a distinct composition of xylose, fucose, and amino sugars, combining interfacial functionality with intrinsic bioactivity. Comprehensive characterization was performed using spectroscopic (FTIR, ^1^H NMR and ^1^H–^1^H COSY NMR), chromatographic (GPC, GC–MS), thermal (TGA, DSC), and structural (XRD, SEM) techniques. In addition, rheological behavior, emulsifying performance, and biological activities, including antibacterial, antioxidant, and cytocompatibility responses, were evaluated to assess its multifunctional potential. By integrating compositional analysis with functional performance, this work identifies associations between structural heterogeneity and multifunctional behavior, positioning cyanobacterial EPSs as promising candidates for the development of biobased materials with combined interfacial and bioactive functionalities.

## Materials and Methods

### EPS Isolation from *Euhalothece* sp. AQUAMEB-3

The cyanobacterium strain, AQUAMEB-3, was obtained from the Culture Collection of Microalgae and Cyanobacteria at Bursa Technical University (https://www.aquameb.org/shop). Cultures were maintained and scaled up in 60 ppt salinity Plymouth Erd-Schreiber (PES) medium, prepared in-house according to the established formulation^25^ which has also been employed for the cultivation of halotolerant microalgae under elevated salinity conditions,^26^ under a 12 h:12 h light–dark cycle at an irradiance of 100 μmol photons m^–2^ s^–1^ and a temperature of 23 °C until reaching the late stationary phase. EPS production was carried out in a total culture volume of 5 L.

Cells were removed by centrifugation at 3000*g* for 15 min at 4 °C, and the supernatant containing extracellular polymeric substances (EPS) was carefully collected. EPS was precipitated by adding two volumes of cold (4 °C) 95% ethanol and incubating the mixture overnight at 4 °C to ensure complete precipitation.

The precipitated EPS was collected using a metal filter and transferred into a dialysis membrane with a molecular weight cutoff of 14 kDa (Sigma-Aldrich, USA). Dialysis was first performed against a minimum of 10 volumes of 0.5 M EDTA solution for 24 h, followed by dialysis against distilled water for 24 h under continuous stirring, with periodic water replacement to ensure efficient removal of residual salts and low-molecular-weight impurities.^27^

The purified EPS was subsequently freeze-dried and stored at room temperature in a desiccator until further analysis. This procedure represents a partial purification method without deproteinization or chromatographic fractionation.

### Biochemical Composition of EPS

The biochemical composition of *Euhalothece* sp. AQUAMEB-3 EPS was determined using colorimetric methods. Total carbohydrate content was determined by the phenol–sulfuric acid method.^28^ EPS samples (0.5 mg mL^–1^; 1 mL) were mixed with 3 mL of concentrated sulfuric acid, vortexed for 30 s, and supplemented with 1 mL of 5% (w/v) phenol. After incubation at 90 °C for 5 min, absorbance was measured at 490 nm using d-glucose as the standard.

Total protein content was determined using a modified Lowry method.^29^ Lyophilized EPS (1 mg) was treated with 40 μL of 24% (w/v) trichloroacetic acid at 95 °C for 15 min, diluted with 120 μL of distilled water, and centrifuged at 15,000*g* for 20 min at 4 °C. The pellet was mixed with 100 μL of Lowry reagent D [A/B/C, 48:1:1; A, 2% (w/v) Na_2_CO_3_ in 0.1 N NaOH; B, 1% (w/v) sodium potassium tartrate tetrahydrate; C, 0.5% (w/v) CuSO_4_·5H_2_O], incubated at 55 °C for 3 h, and centrifuged at 15,000*g* for 20 min. For quantification, 10 μL of the resulting extract was mixed with 190 μL of Lowry reagent D for 10 min, followed by 20 μL of 1:1 diluted Folin–Ciocalteu reagent. Absorbance was measured at 600 nm after 30 min using BSA as the standard.

Uronic acid content was determined by the *m*-hydroxydiphenyl method.^30^ EPS (1 mg mL^–1^; 200 μL) was mixed with 1.2 mL of 0.0125 M sodium tetraborate in concentrated sulfuric acid and heated at 100 °C for 5 min. After cooling on ice, 20 μL of *m*-hydroxydiphenyl reagent [0.15% (w/v) in 0.5% (w/v) NaOH] was added, and absorbance was measured at 520 nm within 5 min using galacturonic acid as the standard.

Neutral sugar content was determined using the resorcinol method.^31^ EPS solutions (1 mg mL^–1^) were diluted 5-fold, and 200 μL of the diluted sample was mixed with 200 μL of resorcinol solution and 1 mL of 75% sulfuric acid. Absorbance was measured at 430 nm using d-galactose as the standard.

For comparison of chemical composition, each culture condition was evaluated using independent day-to-day biological experiments (log phase/150 ppt PES, *n* = 3; late stationary/60 ppt PES, *n* = 2), with technical triplicates performed within each experiment.

### Fourier Transformed Infrared (FTIR) Spectroscopy of EPS Samples

From here-on, all analyses were performed on EPS obtained from a late stationary 60 ppt PES culture. The functional group analysis of EPS samples was performed using a Spectrum Two FTIR spectrophotometer (PerkinElmer, Massachusetts, U.S.A). The freeze-dried EPS was analyzed in powder form using attenuated total reflectance (ATR) mode, with the sample placed directly on the ATR crystal. The FTIR spectrum was measured within the spectral range of 400–4000 cm^–1^, with a data resolution of 4 cm^–1^.^32^

### Thermogravimetric Analysis (TGA) of EPS

The thermal stability of the lyophilized EPS was investigated using the Ta/SDT650 thermal gravimetric analyzer (New Castle, USA). TGA was performed within the temperature range of 25–800 °C under a nitrogen atmosphere at a flow rate of 100 mL min^–1^ and a heating rate of 10 °C min^–1^.

### Differential Scanning Calorimetry (DSC) Analysis of EPS

The DSC analysis was carried out using a TA Instruments DSC250 differential scanning calorimetry (New Castle, USA). The lyophilized EPS was used and the DSC analysis was performed within the temperature range of 0–200 °C, with a flow rate of 5 mL min^–1^ under a nitrogen atmosphere.

### X-ray Diffraction (XRD) Analysis of EPS

The XRD patterns were obtained using a Bruker D8-Discover diffractometer, with Cu Kα radiation, a step size of 0.02°, and a dwelling time of 0.5 s. The freeze-dried EPS was analyzed in powder form and placed directly onto the sample holder for XRD measurement. The crystallinity index (CI_XRD_) was determined by calculating the area under the crystalline peaks and normalizing it with respect to the total scattering area using the following equation^33^

$${CI}_{XRD}=\frac{\sum A_{crystal}}{\sum A_{crystal}+\sum A_{amorphous}}$$

### Scanning Electron Microscopy (SEM) Analysis of EPS

The morphological properties of freeze-dried *Euhalothece* sp. AQUAMEB-3 EPS was examined with SEM using Zeiss Gemini SEM 300. EPS samples prepared at concentrations of 2%, 1%, and 0.5% in deionized water were lyophilized to preserve their three-dimensional porous structures, then coated with a thin Au–Pd layer (15 nm) and imaged with SEM at different magnifications.

### Determination of Zeta Potential of EPS

The zeta potential of the *Euhalothece* sp. AQUAMEB-3 EPS was determined using dynamic light scattering (DLS) with a Zetasizer Nano ZS instrument (Malvern Instruments, CO., UK) to complement the information on the ionic charge of the EPS. The measurement of EPS (0.5% w/v) in deionized water was carried out at a temperature of 25 °C. The values were calculated as the mean of three independent measurements.^34^

### Molecular Mass Determination of EPS

Gel permeation chromatography (GPC) was used to determine the molecular weight distribution and average molecular weights of the EPS sample using an Agilent Technologies 1260 Infinity system equipped with a PL aquagel-OH MIXED-H column (300 mm length × 7.5 mm internal diameter, 8 μm particle size) and refractive index and viscometric detectors. EPS samples were prepared at a concentration of 0.5% (w/v) in deionized water. The analysis was performed at 30 °C using phosphate-buffered saline (PBS, pH 7.0) containing 0.05% NaN_3_ as the mobile phase at a flow rate of 1 mL min^–1^. Molecular weights were determined using viscosity-based universal calibration with linear poly­(ethylene oxide) (PEO) standards with peak molecular weights (*M*_p_) ranging from 109 to 1,522,000 g mol^–1^.

### Determination of Monosaccharide Composition of EPS

#### EPS Hydrolysis

500 μL of 2 M HCl was added to 1 mg of dry EPS samples. The following monosaccharide standards were used for structural comparison throughout the study: xylose, fucose, fructose, glucosamine, *N*-acetylglucosamine (NAG), arabinose, rhamnose, mannose, talose, galactose, glucuronic acid, ribose, 2-deoxyribose, alose, and lyxose. In addition, *N*-acetylglucosamine (NAG), glucosamine, and glucuronic acid standards were subjected to the same acid hydrolysis conditions (500 μL of 2 M HCl, 105 °C for 2 h) to evaluate their behavior under the hydrolysis conditions used for the EPS samples. The acid was removed under a gentle nitrogen stream at 70 °C.

### Derivatization of Hydrolyzed EPS

150 μL of methoxyamine hydrochloride (10 mg/mL; in pyridine) was added to the hydrolyzed EPS, NAG and glucuronic acid, and to 0.5 mg of nonhydrolyzed sugar standards. The samples and standards were incubated for 90 min at 40 °C to form the corresponding imines. 150 μL of 1% trimethylchlorosilane in *N*-methyl-*N*-(trimethylsilyl)­trifluoroacetamide was added and all samples and standards were incubated at 50 °C for 30 min.

### GC–MS Analysis of Hydrolyzed EPS

GC–MS-analysis was performed with a 5977B mass selective detector (Agilent, Santa Clara, California, USA) equipped with a (5%-phenyl)-methylpolysiloxane capillary column (HP-5MS; 30 m × 0.25 mm, 0.25 μm film thickness; Agilent, Santa Clara, California, USA) and a quadrupole detector working with electron impact ionization at 70 eV. Aliquots of the derivatized samples and standards were injected in 1:5 split mode. The interface temperature was 250 °C and the helium inlet pressure was 12.93 psi. After sample injection, the column was first kept at 100 °C for 2 min and then heated with a temperature gradient of 7 °C/min to a final temperature of 310 °C. Analysis was done with MassHunter Qualitative Analysis 10.0 software (Agilent, Santa Clara, California, USA). Monosaccharides were identified by comparing retention times to those of standards. Amino acids were identified with NIST 2.3 library (National Institute of Standards and Technology, Gaithersburg, Maryland, USA).

### ^1^H NMR and ^1^H–^1^H COSY NMR Analysis of Hydrolyzed EPS

Hydrolyzed EPS samples were freeze-dried and the residues were dissolved in 0.5 mL D_2_O. The spectra were recorded at 27 °C with a Bruker Avance III 500 MHz spectrometer. The instrument was equipped with a 5 mm SEI probe. The spectrometer was operated with TopSpin Version 2.1 (Bruker Biospin GmbH, Rheinstetten, Germany). One dimensional ^1^H NMR spectra were recorded with suppression of the residual signal of HDO, two-dimensional ^1^H–^1^H COSY NMR spectra were recorded using standard parameter sets of the Bruker software. The spectra were processed with MestreNova Version 15.0.0 (Mestrelab Research, Santiago de Compostela, Spain). The ^1^H NMR and ^1^H–^1^H COSY NMR signals were assigned on the basis of the observed correlations in the two-dimensional spectra and by comparison of reference spectra.

### Rheological Property Analysis of EPS

Viscosity measurements of EPS solutions (0.1%, 0.5%, 1%) prepared in deionized water were performed by varying the shear rates (0.01–1000 s^–1^) at 25 °C in rotational rheometer C-PTD 180/AIR/QC (Anton Paar). The solutions containing 0.1%, 0.5%, 1% xanthan gum (MW > 2000 g mol^–1^, MP Biomedicals, LLC., CA, USA) were used as standards.^35^

### Emulsifying Properties of EPS

The emulsifying capacity of *Euhalothece* sp. AQUAMEB-3 EPS was determined using the method as previously described.^36^ In flat bottom glass tubes, 3 mL of liquid paraffin (Merck, Germany) was mixed with 2 mL of 0.1%, 0.5%, 1.0% (w/v) *Euhalothece* sp. AQUAMEB-3 EPS aqueous solutions and stirred vigorously in a vortex for 2 min. The emulsion index (*E*_24_) was calculated after being kept at room temperature for 24 h. Xanthan gum solutions at the same concentrations were utilized as standard solutions. All tests were carried out with three replicates. The emulsion index (*E*_24_) was calculated using the following equation

$$E_{24}=\frac{h_{e}}{h_{T}}\times 100$$

where *h*_e_ represents the height of the emulsion layer in millimeters and *h*_T_ is the total height of the mixture in millimeters.^35^ The microscopic observations of the emulsions were performed with a Leica BM2500 polarized light microscope using a ×50 objective lens.

### 1,1-Diphenyl-2-picrylhydrazyl (DPPH) Radical-Scavenging Assay of EPS

The DPPH radical scavenging capacity assay was carried out with the modified method as previously described, with slight modifications.^37^ In this method, 17 μL of methanolic DPPH solution (1 mM) was added to 133 μL of aqueous EPS solution. The control contained 133 μL of water and 17 μL of 1 mM DPPH solution without EPS. Ascorbic acid was used as a positive reference control and was tested over the same concentration range and under the same assay conditions as the EPS. For each EPS concentration, a sample blank containing the corresponding EPS solution and methanol without DPPH was prepared to correct for intrinsic absorbance and light scattering. At the end of the incubation period, the absorbance of the samples and corresponding sample blanks was measured at 517 nm, and the radical scavenging activity was calculated as percentage inhibition using the following equation

$$Inhibition\;\left(\%\right)=\frac{A_{control}-\left(A_{sample}-A_{blank}\right)}{A_{control}}\times 100$$

where *A*_control_ is the absorbance of the DPPH control without EPS, *A*_sample_ is the absorbance of the EPS–DPPH reaction mixture, and *A*_blank_ is the absorbance of the corresponding EPS sample without DPPH.

### Chelating Effects of EPS on Ferrous Ions (Fe^2+^)–Ferrozine Assay of EPS

The estimation of ferrous ion chelation was performed using a microplate-adapted method.^38^ In this method, 200 μL of *Euhalothece* sp. AQUAMEB-3 EPS sample was mixed with a solution containing 10 μL of ferrous chloride at a concentration of 2 mM. The reaction began by adding 40 μL of ferrozine (5 mM) and incubating it at room temperature for 10 min. Subsequently, the absorbance was determined at a wavelength of 562 nm. EDTA was used as a positive control, as previously reported.^38^ The blank solution consisted of water and FeSO_4_·7H_2_O, while the control solution consisted of FeSO_4_·7H_2_O and ferrozine. The calculation of the inhibition percentage of ferrozine–Fe^2+^ complex formation was performed using the following equation.^39^

$$Iron\;chelation\;\left(\%\right)=\frac{A_{control}-A_{sample}}{A_{control}}\times 100$$

where *A*_control_ is the absorbance of the control and *A*_sample_ is the absorbance of the sample.

### Antibacterial Activity of EPS

The antibacterial activity of *Euhalothece* sp. AQUAMEB-3 EPS was determined as the minimum inhibitory concentration (MIC) using the broth microdilution method.^40^ *Escherichia coli* (ATTC 25922) and *Staphylococcus aureus* (ATTC 25923) bacterial cultures were incubated on agar plates at 37 °C for 24 h and subsequently inoculated into nutrient broth (Merck, Germany). Before the assay, the turbidity of the microbial suspension was calibrated to a McFarland standard of 0.5, corresponding to concentrations of 1–5 × 10^8^ CFU/mL for each bacterium. The microbial suspensions were subsequently diluted (1:100, v/v) with nutrient broth and used in the assays below. The method described by Eloff (1998) was used for determining the minimum inhibitory concentration (MIC) values of *Euhalothece* sp. AQUAMEB-3 EPS against bacterial strains. A 100 μL volume of the *Euhalothece* sp. AQUAMEB-3 EPS (5000 μg mL^–1^) dissolved in deionized water was serially diluted 2-fold in triplicates using sterile deionized water in 96-well microtiter plates, to which 30 μL of bacterial cultures in nutrient broth was added in each well. Nutrient broth was added to achieve a total well volume of 200 μL. The tested EPS concentrations ranged from 39 μg mL^–1^ to 2500 μg mL^–1^. Ampicillin and deionized water were used as positive and negative controls, respectively. Plates with test bacteria were incubated for 24 h at 37 °C. To visualize the growth of bacteria, 40 μL of 0.2 mg mL^–1^ *p*-iodonitrotetrazolium violet (INT) (Sigma-Aldrich, USA.) was added to each well at the end of the incubation period. Plates were further incubated for 1–2 h and the color of INT changed from clear to purple in case there was bacterial growth.^41^ The minimum inhibitory concentration (MIC) was defined as the lowest concentration of EPS that reduced bacterial growth after 24 h.

### Cytotoxicity Assessment of EPS

The cytotoxicity of the EPS was evaluated using the 3-(4,5-dimethylthiazol-2-yl)-2,5-diphenyltetrazolium bromide (MTT) assay in accordance with ISO 10993-5 guidelines. L929 mouse fibroblasts, BEAS-2B human bronchial epithelial cells, and A549 human lung carcinoma cells were cultured under standard conditions (37 °C, 5% CO_2_, 95% humidity) in RPMI 1640 medium supplemented with 10% fetal bovine serum and 1% penicillin–streptomycin.

Cells were seeded into 96-well plates at a density of 1 × 10^4^ cells per well (200 μL) and incubated for 24 h to allow attachment. Subsequently, the culture medium was replaced with EPS solutions prepared in sterile water at different concentrations (5–2000 μg mL^–1^). Fresh culture medium and medium containing 1% Triton X-100 were used as negative and positive controls, respectively. Cells were further incubated for 24 h.^42^

After treatment, the medium was removed and each well was supplemented with 100 μL fresh medium and 10 μL MTT solution. Following 4 h incubation, the formed formazan crystals were dissolved in 100 μL DMSO, and absorbance was measured at 570 nm using a microplate reader. Cell viability (%) was calculated relative to the negative control. All experiments were performed in triplicate (*n* = 3).

### Statistical Analysis

All experiments were conducted using independent biological replicates as specified for each data set. Within each experiment, measurements were performed in technical triplicates, and the mean of technical replicates was used as a single observation for statistical analysis to avoid pseudoreplication.

Data are presented as mean ± standard deviation (SD). For comparisons between two experimental conditions with small sample sizes (e.g., *n* = 3 vs *n* = 2), Welch’s two-sample *t*-test was applied to account for unequal variances. To ensure robustness, statistical significance was further validated using two-sided permutation tests.

For comparisons involving more than two groups under a single factor (e.g., concentration-dependent assays such as ferrous ions chelating and antioxidant activity), one-way analysis of variance (ANOVA) followed by Tukey’s post hoc test was performed when assumptions of normality and homogeneity of variance were satisfied.

For factorial experimental designs involving two independent variables (e.g., polymer type and concentration in emulsification studies), two-way ANOVA was applied to evaluate main effects and interaction effects. When significant differences were detected, Tukey’s honestly significant difference (HSD) test was used for multiple comparisons.

A *p*-value <0.05 was considered statistically significant. All statistical analyses were performed using SPSS version 25.0 (IBM Corp., Armonk, NY, USA).

## Results and Discussion

### Biochemical Composition of EPS

The EPS obtained from *Euhalothece* sp. AQUAMEB-3 appeared as a white, amorphous material (Figure 1) with a slightly acidic pH (5.95 ± 0.1 at 5 g L^–1^), likely attributable to the presence of uronic acid residues. This amorphous structure is advantageous for functional applications, as it facilitates molecular interactions and enhances accessibility of active binding sites.

**Figure 1:**
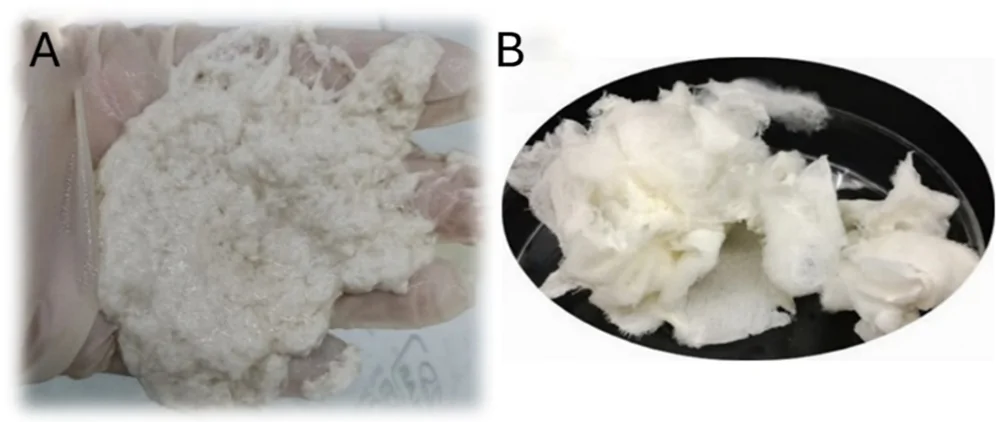
Photos of isolated exopolysaccharide (EPS) from *Euhalothece* sp. AQUAMEB-3 (A) EPS after precipitation with ethanol, (B) lyophilized EPS.

Biochemical composition and EPS yield were evaluated using independent cultivation runs as biological replicates (log phase/150 ppt PES, *n* = 3; late stationary/60 ppt PES, *n* = 2) (Table 1). No statistically significant differences were observed between the two conditions for protein (Welch’s *t*-test, *p* = 0.778), carbohydrate (*p* = 0.451), or uronic acid contents (*p* = 0.485), indicating that the macromolecular composition of the EPS remained stable across growth phase and salinity conditions. Such compositional robustness is advantageous for reproducible functional performance.

**Table 1:** Comparison of Biochemical Composition and Exopolysaccharide (EPS) Yield for Cultures in Log-Phase/150 ppt vs Late-Stationary Phase/60 ppt^a^ ^,^ ^b^

	protein (mg g^–1^ DW)	carbohydrate (mg g^–1^ DW)	uronic acid (mg g^–1^ DW)	EPS (mg L^–1^ DW)
log phase/150 ppt PES	39.60 ± 11.71	757.63 ± 141.79	66.49 ± 9.07	118.33 ± 17.47
late stationary/60 ppt PES	43.25 ± 12.94	625.05 ± 164.83	55.56 ± 15.25	889.00 ± 213.55

a Values are presented as mean ± SD of independent experiments (*n* = 3 for log phase/150 ppt PES; *n* = 2 for late stationary/60 ppt PES). Each experiment was also performed in technical triplicates.

b DW, dry weight.

In contrast, the mean EPS yield increased from 118.33 ± 17.47 mg DW L^–1^ in log-phase cultures to 889.00 ± 213.55 mg DW L^–1^ in late stationary-phase cultures. However, this difference was not statistically significant (*p* = 0.122), likely because of the limited and unequal number of independent biological replicates. Therefore, the observed increase should be interpreted as a preliminary trend rather than conclusive evidence of enhanced EPS production. Nevertheless, this trend is consistent with previous reports describing increased EPS synthesis under stress conditions and during late growth stages.^43^

The EPS yield obtained in this study falls within the range reported for cyanobacterial systems, particularly those derived from halotolerant strains.^43^ Reported EPS production by cyanobacteria varies considerably depending on the producing strain and cultivation conditions. Previous studies have reported soluble EPS productivities of approximately 115 mg L^–1^ day^–1^ ^44^ for *Cyanothece* sp. under nitrogen starvation, whereas the freshwater cyanobacterium *Nostoc* *cf.* *linckia* produced 5.4 g L^–1^ crude EPS.^45^ More broadly, cyanobacterial EPS production is known to vary substantially among species and cultivation strategies, highlighting the strong influence of physiological and environmental factors on productivity.^46,47^ The comparatively lower yield observed for *Euhalothece* sp. AQUAMEB-3 may reflect lack of process optimization as well as the metabolic cost associated with the synthesis of structurally complex heteropolysaccharides.

EPS production in cyanobacteria is known to be influenced by environmental and physiological parameters, including temperature, light intensity, nutrient availability, and aeration.^48^ Since no systematic optimization was performed in this study, the reported values may not fully reflect the maximum production capacity of this strain.

The relative proportions of neutral sugars and uronic acids in AQUAMEB-3 EPS are consistent with the heterogeneous composition typically reported for cyanobacterial EPSs.^6,49^ Previous studies have demonstrated considerable variation in carbohydrate and protein contents among cyanobacterial EPSs. For example, carbohydrate contents ranging from approximately 56–87% and protein contents of 1.8–4.0% have been reported for EPSs from *Oscillatoria* sp., *Nostoc* sp., *Nostoc carneum*, and *Cyanothece* sp.^50^ Similarly, the crude EPS produced by *Anabaena* sp. PCC 7120 contained 67.6% carbohydrate and 23.9% protein,^51^ whereas the alginate-like EPS from *Synechocystis algini* MNE ON864447 contained approximately 70–75% carbohydrate and only 0.9% protein.^43^ In contrast, the EPS of *Arthrospira platensis* contained 13% carbohydrate and 55% protein.^52^ These differences reflect variations in purification strategies, cultivation conditions, and organism-specific metabolism.

The EPS isolation procedure used in this study, based on ethanol precipitation and dialysis, represents a partial purification approach.^43^ Consequently, the detected protein fraction (Table 1) may include both loosely associated extracellular proteins and structural components of the EPS matrix. Given the partial purification procedure used in this study, the detected protein fraction may comprise proteins intrinsically associated with the EPS matrix as well as residual extracellular proteins that remained associated during isolation. Therefore, the extent to which the protein fraction contributes to the observed biological and interfacial properties cannot be determined from the present data. Whether these proteins are covalently bound to the polysaccharide or associated through noncovalent interactions also remains unclear.^45^ Future studies employing chromatographic fractionation, further purification, and selective protein-removal approaches could help resolve individual EPS-associated components and clarify the respective contributions of the polysaccharide and protein fractions to the observed functional properties. Accordingly, the functional properties reported in the present study should be interpreted as those of the partially purified EPS preparation rather than of a completely purified polysaccharide fraction. Future studies employing dedicated deproteinization procedures and comparative analyses of purified and deproteinized EPS fractions will be necessary to determine the specific contribution of proteinaceous components to the observed antioxidant, antibacterial, and emulsifying properties.

The presence of uronic acids introduces negatively charged carboxyl groups along the polymer backbone, which play a key role in governing the physicochemical behavior of the EPS. These groups facilitate electrostatic interactions and complexation with metal ions, thereby supporting the metal-binding and sorption capacity of the material.^53^ In addition, the hydrophilic nature of these residues enhances water affinity, which is beneficial for applications involving aqueous systems.^54^

Furthermore, the coexistence of hydrophilic polysaccharide components and hydrophobic moieties, such as peptide-derived structures or deoxy sugars, suggests an amphiphilic architecture. This dual character promotes interfacial activity, enabling stabilization of oil–water systems and supporting emulsification, adhesion, and biofilm-related processes.^55^

Overall, the compositional and structural characteristics of AQUAMEB-3 EPS, including its anionic nature, macromolecular complexity, and amphiphilic properties, collectively support its potential as a multifunctional biomaterial. These features are particularly relevant for applications such as metal ion removal, emulsion stabilization, and bioactive formulations.^56,57^

### FTIR Spectroscopy of EPS

The Fourier-transform infrared (FTIR) spectrum of *Euhalothece* sp. AQUAMEB-3 EPS (Figure 2) revealed characteristic absorption bands consistent with a structurally complex heteropolysaccharide, in agreement with GC–MS and ^1^H–^1^H COSY NMR analyses. The broad band at 3330 cm^–1^ corresponds to O–H stretching vibrations of hydroxyl groups, a defining feature of carbohydrate-rich polymers.^43^ The abundance of hydroxyl functionalities may be associated with hydrogen-bonding capacity and the hydrophilic character of the EPS.

**Figure 2:**
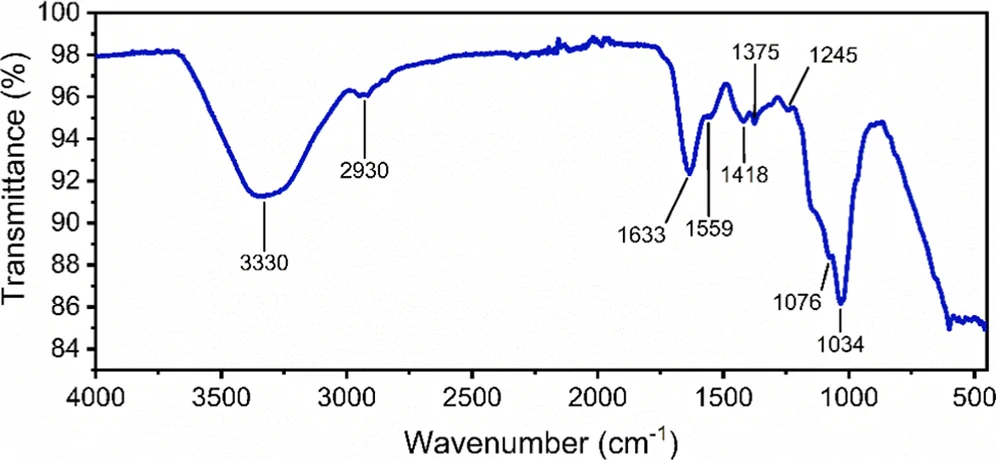
Fourier transformed infrared (FTIR) spectrum of *Euhalothece* sp. AQUAMEB-3 exopolysaccharide (EPS). The arrows indicate the main absorption bands and their corresponding wavenumbers.

The weak doublet around 2930 cm^–1^ is attributed to C–H stretching vibrations of −CH and −CH_2_ groups in sugar residues.^58^ These aliphatic moieties introduce limited hydrophobic character, supporting the amphiphilic nature of the EPS and its potential to stabilize oil–water interfaces in emulsification systems.

A prominent band centered at 1633 cm^–1^, with a shoulder at 1559 cm^–1^, is assigned to asymmetric stretching of carboxylate groups (COO^–^) and possible amide II vibrations, respectively. These features are consistent with carboxylate- and amide-related functionalities and are in agreement with the complementary compositional analyses.^59,60^

Additional bands at 1418 cm^–1^ and 1375 cm^–1^ correspond to symmetric COO^–^ stretching and C–H bending vibrations, likely associated with methyl and methylene groups in deoxy sugars such as fucose.^61^ These structural elements further support the presence of heterogeneous sugar residues contributing to both structural flexibility and interfacial activity.

A smaller band at 1245 cm^–1^ is attributed to C–O–C stretching vibrations of glycosidic linkages, indicative of a polysaccharide backbone with interconnected sugar units. The fingerprint region (1200–800 cm^–1^) exhibits multiple bands characteristic of sugar ring vibrations and glycosidic linkages.^58^ In particular, the broad band centered at 1034 cm^–1^, with shoulders at 1150 and 1076 cm^–1^, corresponds to C–O–C and C–O stretching, ring deformation, and conformational flexibility. These features are associated with pentose, deoxy, and amino sugars such as xylose, fucose, and glucosamine.

Overall, the FTIR profile indicates the presence of diverse functional groups in AQUAMEB-3 EPS, including hydroxyl, carboxylate, amide-related, and glycosidic functionalities. Together with the complementary compositional and functional analyses, these features may be associated with the physicochemical and biological properties observed for the EPS.

### DSC Analysis of EPS

Differential scanning calorimetry (DSC) was employed to investigate the thermal transitions of the EPS and to assess its suitability for processing and functional applications.^62^ The DSC thermogram (Figure 3A) exhibited a single broad endothermic peak centered at 86.5 °C, which is attributed to the desorption of bound water and/or a glass transition rather than a crystallization event. This observation is consistent with the 17% weight loss detected by TGA up to 90 °C, confirming the presence of significant amounts of physically and chemically bound moisture in the polymer matrix.^63^

**Figure 3:**
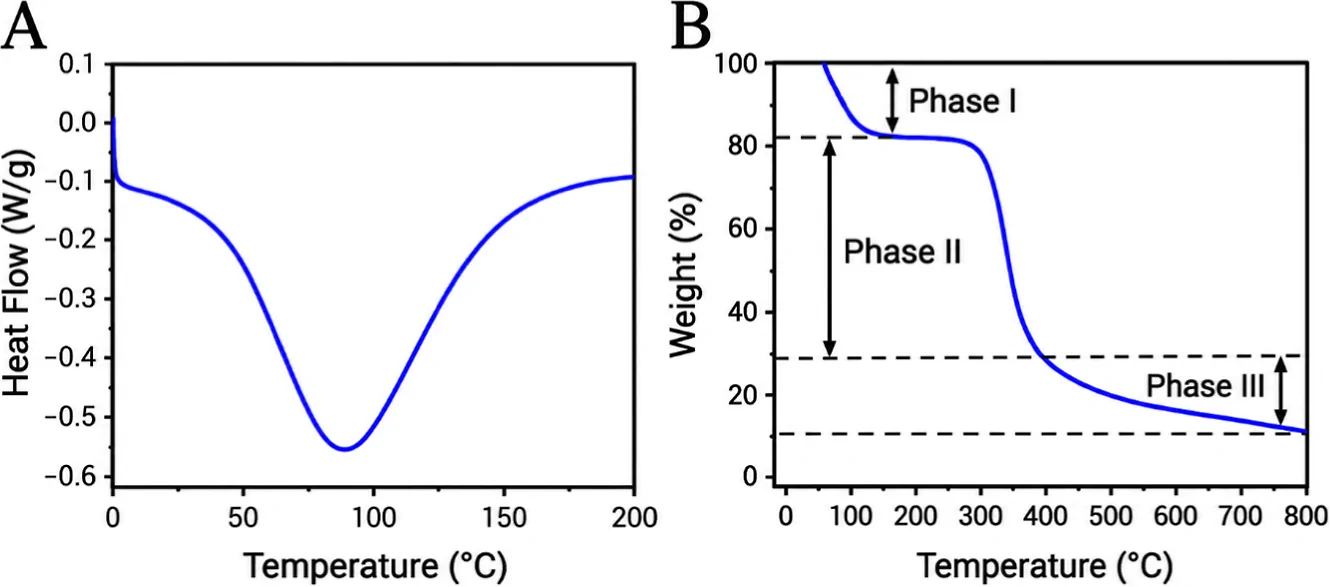
(A) Differential scanning calorimetry (DSC) diagram. (B) Thermogravimetric analysis (TGA) of *Euhalothece* sp. AQUAMEB-3 exopolysaccharide (EPS).

The absence of distinct melting or crystallization peaks within the 0–200 °C range is in agreement with the low crystallinity index (0.26) obtained from XRD analysis, indicating that the EPS possesses a predominantly amorphous structure.^64^ Such amorphous organization facilitates molecular mobility and contributes to softening behavior upon heating, which is advantageous for processing and interfacial applications.^33^

From a functional perspective, the strong water-binding capacity observed in the DSC profile is consistent with the high content of hydroxyl and uronic acid groups identified by FTIR. These hydrophilic functionalities promote moisture retention and enhance polymer–water interactions, which are critical for emulsification, stabilization of dispersed systems, and bioactive performance in aqueous environments.

Furthermore, the absence of sharp thermal transitions suggests that the EPS maintains structural integrity under moderate thermal conditions, which is beneficial for preserving bioactive functionalities such as antioxidant and antibacterial activity during processing. This behavior supports the potential use of AQUAMEB-3 EPS in temperature-sensitive formulations, including food, pharmaceutical, and biomedical applications.

### TGA Analysis of EPS

Thermogravimetric analysis (TGA) was conducted to evaluate the thermal degradation behavior of *Euhalothece* sp. AQUAMEB-3 EPS from 25 to 800 °C under nitrogen atmosphere (Figure 3B). The thermogram revealed three distinct degradation stages, which are characteristic of cyanobacterial polysaccharides.^33,52,65^

The first stage (25–90 °C) showed approximately 17% mass loss, corresponding to the removal of physically adsorbed and bound water. This behavior reflects the highly hydrophilic nature of the EPS, which is associated with the presence of carboxyl and uronic acid groups.^24^ Such water-retention properties are relevant to the use of microbial EPSs as moisture-retaining, thickening, and stabilizing agents in food and cosmetic formulations, while their hydrocolloidal properties also support their use in emulsion-based systems.^66,67^

The second stage (243.4–347.5 °C) exhibited a major mass loss of 51.5%, attributed to the depolymerization of the polysaccharide backbone through cleavage of glycosidic linkages and degradation of constituent sugars such as fucose and xylose. This process is consistent with the structural features identified by FTIR, particularly the presence of C–O–C and COO^–^ functional groups. The degradation pattern indicates that the EPS matrix is thermally stable up to approximately 243.4 °C.

The third stage (>347.5–800 °C) resulted in an additional mass loss of 17.5%, corresponding to the formation and gradual degradation of thermally stable carbonaceous residues. This behavior is typical for complex heteropolysaccharides containing amino and deoxy sugars, which contribute to char formation during thermal decomposition.

Overall, the thermal stability of AQUAMEB-3 EPS can be attributed to its chemically diverse structure, including uronic acids and amino sugars, which may retard complete degradation.^43^ The observed degradation profile follows typical polysaccharide decomposition pathways, involving dehydration, depolymerization, and formation of stable carbonaceous structures.^50^

Importantly, when considered together with DSC results, the thermal behavior of the EPS indicates a balance between processability and stability. The observed thermal profile indicates that major thermal decomposition of the EPS occurred above approximately 243 °C, with the principal degradation stage observed between 243.4 and 347.5 °C.

### XRD Analysis of EPS

XRD analysis was conducted to evaluate the structural organization of *Euhalothece* sp. AQUAMEB-3 EPS. The diffractogram (Figure 4) exhibited three broad peaks at 2θ values of 12°, 30°, and 41°, which are characteristic of predominantly amorphous polysaccharide structures, similar to those reported for cyanobacterial EPSs.^33,53,68–70^ In contrast to sharp and well-defined peaks observed in crystalline materials, the broad diffraction patterns indicate a lack of long-range molecular order.

**Figure 4:**
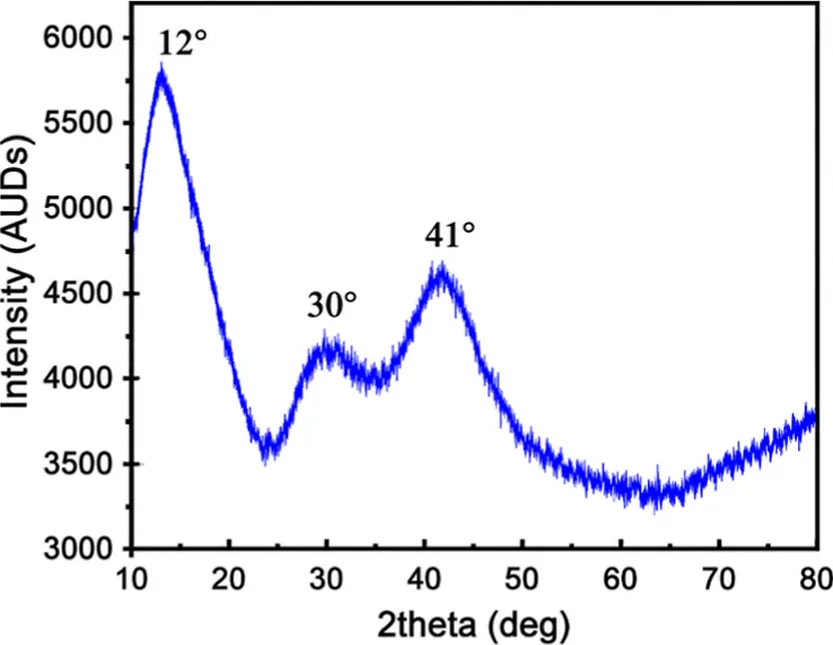
X-ray diffraction (XRD) patterns of *Euhalothece* sp. AQUAMEB-3 exopolysaccharide (EPS).

The crystallinity index (CI_XRD_ = 0.26), calculated as the ratio of crystalline peak area to total scattering area,^33^ further confirms the predominantly amorphous nature of the EPS. This low crystallinity can be attributed to the heteropolymeric composition of the EPS, including irregular monosaccharide units such as xylose and fucose, as well as substituents like uronic acids. These structural irregularities disrupt intermolecular packing and prevent the formation of ordered crystalline domains.^70^

From a functional standpoint, the amorphous structure plays a key role in determining the physicochemical behavior of the EPS. Reduced crystallinity enhances molecular mobility and increases the accessibility of functional groups such as hydroxyl, carboxyl, and amide moieties, as identified by FTIR analysis. This accessibility facilitates interactions with surrounding media, including water molecules, oil phases, metal ions, and microbial cell surfaces.

The observed amorphous profile is comparable to that of biopolymers such as xanthan gum and sodium alginate, although differences in semicrystalline behavior may arise from variations in molecular organization and ionic interactions.^53,71^ In general, amorphous polymers exhibit desirable properties such as flexibility, isotropic behavior, and enhanced interfacial activity,^72^ which are advantageous for applications involving emulsification, adsorption, and bioactive interactions.

Furthermore, the amorphous nature of AQUAMEB-3 EPS complements its thermal behavior (DSC/TGA), where the absence of sharp thermal transitions and the presence of gradual degradation profiles indicate structural heterogeneity and processability. This combination of structural flexibility and thermal stability supports its use in applications requiring controlled morphology, such as encapsulation systems, film-forming materials, and dispersed formulations.^60^

Overall, the XRD results are consistent with a predominantly amorphous structure characterized by limited long-range molecular order. Together with the complementary physicochemical characterization presented in this study, these findings provide structural context for interpreting the rheological, emulsifying, antioxidant, antibacterial, and cytocompatibility properties of AQUAMEB-3 EPS.

### SEM Analysis of EPS

SEM images of lyophilized *Euhalothece* sp. AQUAMEB-3 EPS revealed a porous matrix with interconnected and relatively uniform pore structures (Figure 5). The average pore diameter was 203.52 ± 45.89 μm (*n* = 20), as determined by ImageJ analysis of randomly selected circular and elliptical pores, indicating low structural variability and a well-organized porous network.

**Figure 5:**
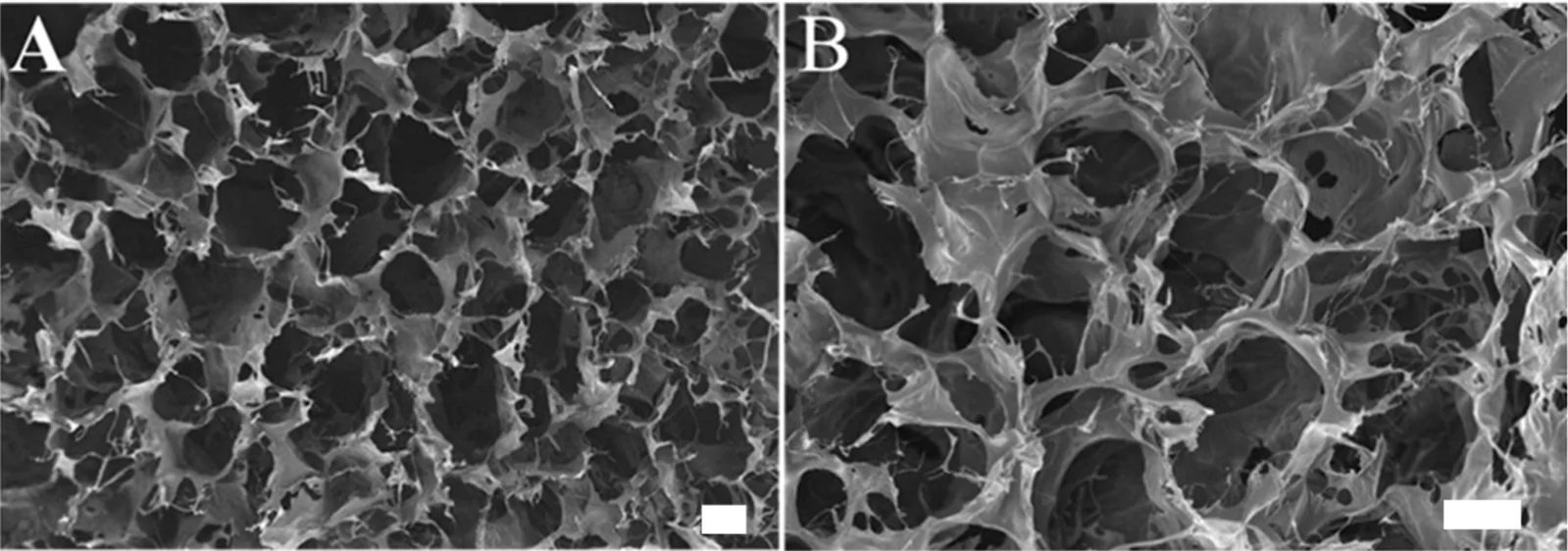
Scanning electron microscopy (SEM) images of lyophilized *Euhalothece* sp. AQUAMEB-3 exopolysaccharide (EPS) (bar = 100 μm) (A) 50× magnification (B) 100× magnification. The scale bar is displayed with a white background to improve its visibility against the SEM micrographs.

This morphology differs from the irregular or flake-like structures reported for certain bacterial EPSs, such as those produced by lactic acid bacteria,^73,74^ and suggests a more homogeneous polymer assembly. The interconnected porous architecture enhances the accessibility of fluids through internal surfaces, which is critical for mass transfer processes. The SEM analysis demonstrates that lyophilized AQUAMEB-3 EPS forms a structurally organized porous network, providing morphological evidence of its interconnected architecture. The combination of interconnected porosity and cohesive polymer organization provides a morphological basis for the physicochemical and functional properties evaluated in the present study, including its rheological behavior and emulsifying capacity.

### Determination of Molecular Weight and Zeta Potential of EPS

GPC analysis of the high-molecular-weight *Euhalothece* sp. AQUAMEB-3 EPS fraction yielded a weight-average *M*_w_ of 2617 kDa (0.5% w/v in deionized water) and a number-average molecular weight (*M*_n_) of 26.25 kDa, corresponding to a dispersity (*M*_w_/*M*_n_) of 99.73. The high dispersity indicates a broad molecular-weight distribution within the analyzed high-molecular-weight fraction.

Molecular weight is an important parameter influencing the rheological and interfacial properties of polysaccharides. Previous studies on microbial EPSs have reported that high-molecular-weight polymers can exhibit high viscosity, pseudoplastic behavior, and useful emulsifying properties. Such rheological and interfacial characteristics support the potential use of microbial EPSs as thickening, stabilizing, and emulsifying agents.^66,67^

The molecular weight of EPS is influenced by genetic background, culture conditions, and growth phase.^75,76^ Molecular-weight variations under different cultivation conditions were not evaluated in the present study.

Zeta potential measurements further provided insight into the colloidal behavior and surface charge characteristics of the EPS. At pH 5.95, the zeta potential of AQUAMEB-3 EPS was determined to be −45.1 ± 2.35 mV, indicating a strongly anionic nature and high colloidal stability.^77^ The strong negative zeta potential (−45.1 mV) indicates a highly anionic character of the EPS, which may be associated with ionizable functional groups within the polymer matrix. Although FTIR analysis suggested the possible presence of phosphate- and sulfate-containing functionalities, these groups were not quantitatively determined in the present study; therefore, their specific contributions to the surface charge cannot be established. In general, ionizable groups in polysaccharides can enhance hydrophilicity and promote electrostatic repulsion, thereby contributing to the stabilization of dispersed systems.^78^

The pronounced anionic character of the EPS plays a central role in its functional behavior. Negatively charged groups facilitate electrostatic interactions and complexation with positively charged species, contributing to its metal-binding capacity and potential application in bioremediation.^79^ At the same time, surface charge and polymer conformation influence interactions with biological systems, including microbial cell membranes.

Overall, the high molecular weight and strong anionic character of AQUAMEB-3 EPS are important physicochemical characteristics that may contribute to its rheological, colloidal, antioxidant, antibacterial, and cytocompatibility properties observed in the present study. These findings provide a basis for future investigations into the potential applications of this EPS in biotechnology and related fields.

### Monosaccharidic Composition of Hydrolyzed EPS

The monosaccharidic composition of *Euhalothece* sp. AQUAMEB-3 EPS was investigated by one-dimensional ^1^H NMR spectroscopy, two-dimensional ^1^H–^1^H COSY NMR spectroscopy, and gas chromatography–mass spectrometry (GC–MS) after acid hydrolysis. The ^1^H NMR spectrum of the EPS hydrolysate (Figure 6A) revealed a complex profile dominated by carbohydrate-derived signals, along with minor contributions from amino acid residues. The expanded sugar region (5.4–2.8 ppm) (Figure 6B) showed multiple overlapping resonances, with the most intense signals observed between approximately 4.5 and 3.5 ppm, consistent with the predominance of carbohydrate-derived signals in the hydrolysate.

**Figure 6:**
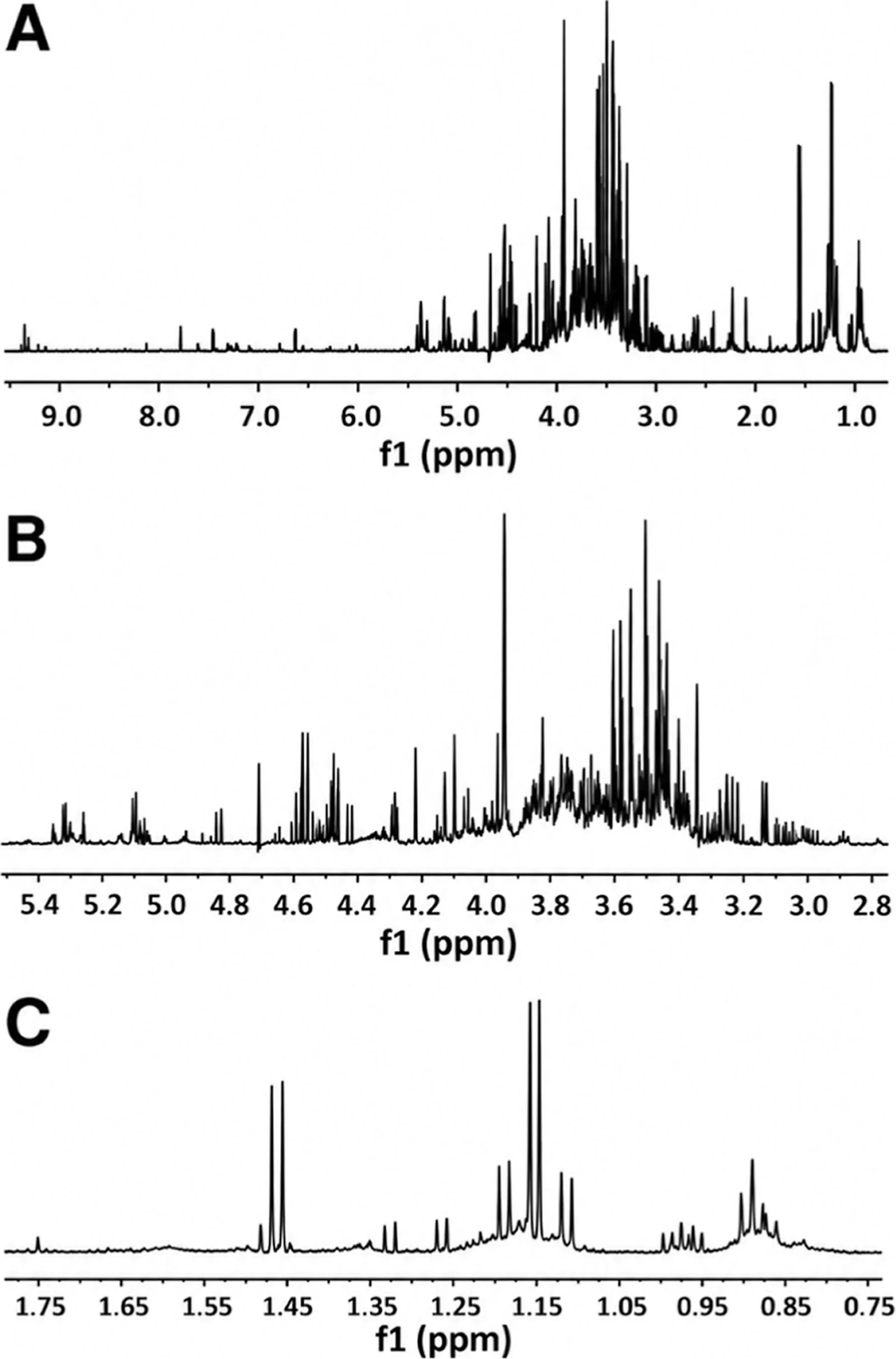
500 MHz proton nuclear magnetic resonance (^1^H NMR) spectrum of hydrolyzed exopolysaccharide (EPS) from *Euhalothece* sp. AQUAMEB-3. (A) Full spectrum, (B) expanded sugar region, and (C) expanded and upscaled view of the high field region indicative of methyl groups.

In the high-field region (<1.75 ppm) (Figure 6C), multiple distinct signals, predominantly doublets, were observed and attributed to methyl groups, indicating the presence of methylated sugars such as deoxyhexoses. In contrast, the low-field region (>5.55 ppm) contained several low-intensity signals, some of which may correspond to aromatic residues (e.g., tyrosine and phenylalanine), suggesting the presence of minor proteinaceous components, in agreement with the biochemical composition results.

Two-dimensional COSY spectra (Figure 7) further supported the presence of methylated sugars, showing characteristic scalar couplings between the high-field (1.5–1.0 ppm) and carbohydrate regions (4.5–3.5 ppm). Comparative analysis with reference monosaccharides indicated that xylose, fucose, and glucose provided the closest spectral matches (Figure 8), although slight variations in chemical shifts were observed due to differences in sample conditions.

**Figure 7:**
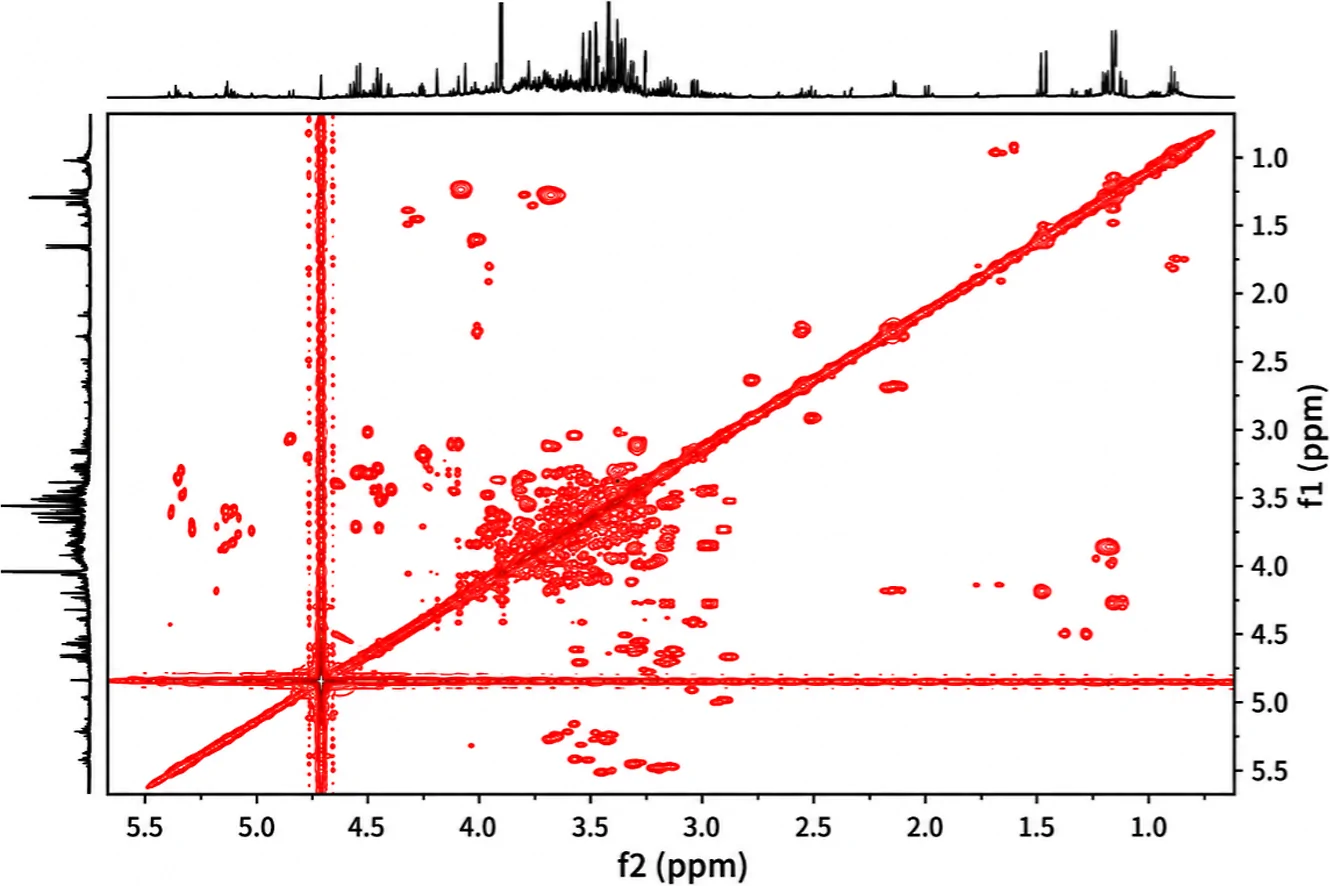
500 MHz correlation spectroscopy (COSY) spectrum of a hydrolyzed AQUAMEB-3 exopolysaccharide (EPS) sample.

**Figure 8:**
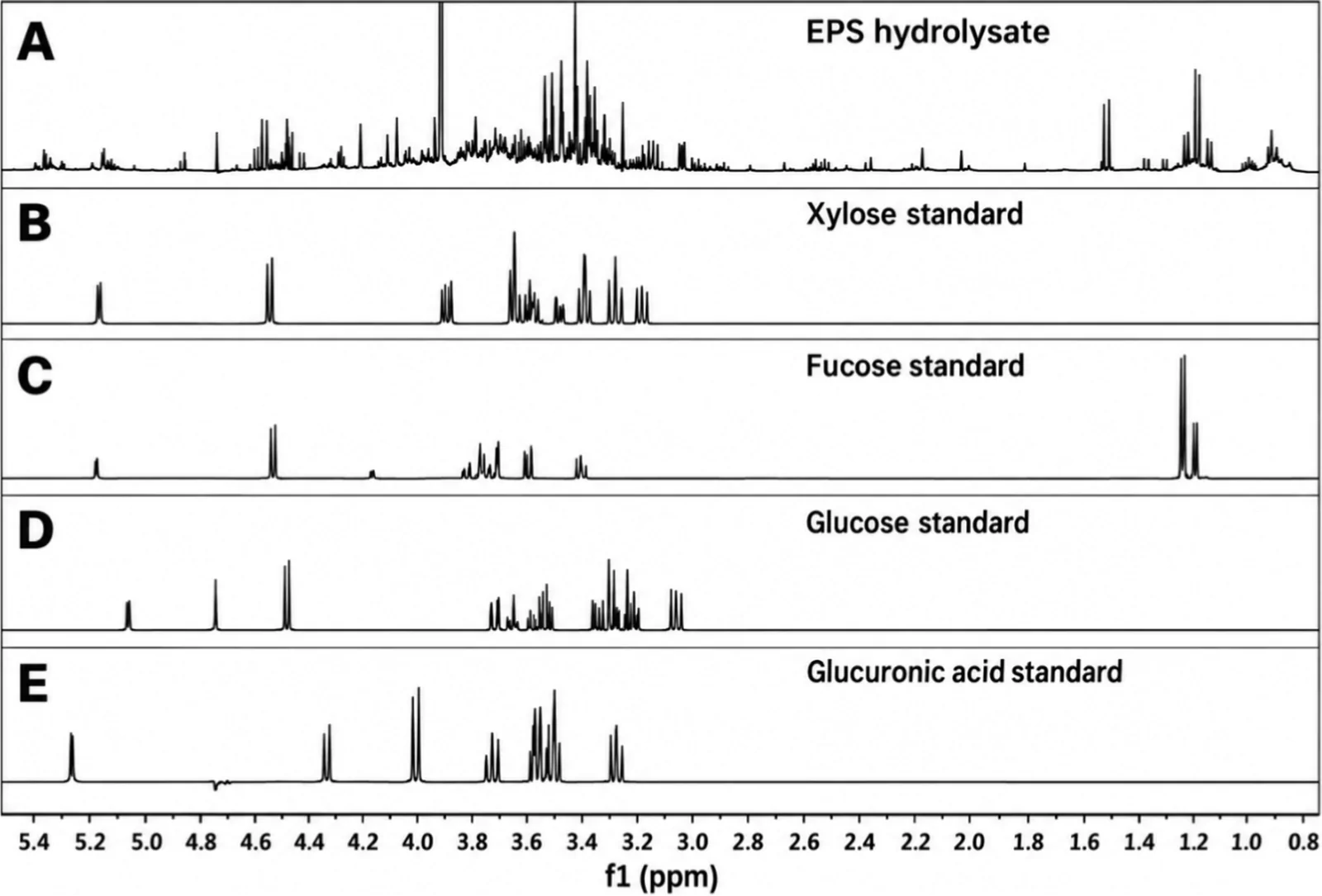
Stacked 500 MHz proton nuclear magnetic resonance (^1^H NMR) spectra of (A) hydrolyzed exopolysaccharide (EPS) from *Euhalothece* sp. AQUAMEB-3 and reference standards: (B) xylose, (C) fucose, (D) glucose, and (E) glucuronic acid.

Overlay analysis of COSY spectra (Figure 9) confirmed strong similarities between the hydrolysate and reference compounds, particularly for α- and β-fucose and xylose, validating their presence as key constituents. Notably, no definitive evidence for glucuronic acid was obtained by ^1^H NMR and ^1^H–^1^H COSY NMR, despite indications from colorimetric assays and FTIR analysis. A glucuronic acid standard subjected to the same acid hydrolysis conditions likewise failed to produce the expected GC–MS derivative, supporting the interpretation that glucuronic acid degrades under the applied hydrolysis conditions.^44^

**Figure 9:**
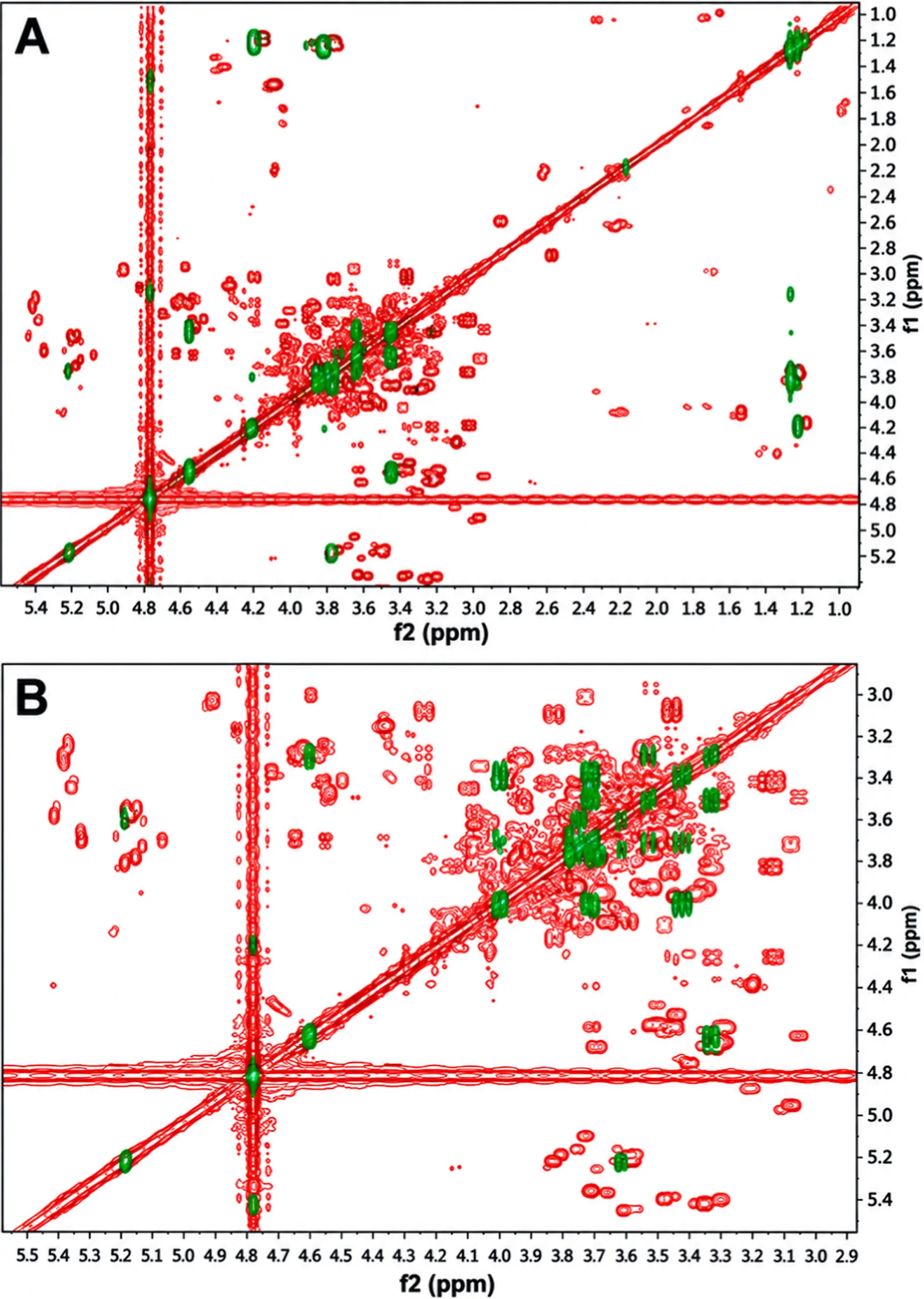
Superimposed 500 MHz ^1^H–^1^H correlation spectroscopy (COSY) NMR spectra of *Euhalothece* sp. AQUAMEB-3 exopolysaccharide (EPS) hydrolysate (signals in red) fucose standard (A) and a xylose standard (B).

GC–MS analysis of derivatized EPS hydrolysates (Figure 10) corroborated the ^1^H NMR and ^1^H–^1^H COSY NMR findings. Xylose and fucose were clearly identified based on retention times and mass spectra, with xylose exhibiting the highest peak intensity, indicating that it is a major monosaccharide component. Fucose was also abundant, albeit at lower levels. Additionally, the amino sugar glucosamine was detected, likely derived partially from hydrolysis of *N*-acetylglucosamine (NAG). Minor amounts of amino acids (Ala, Gly, and Asp) were also identified, consistent with their lower abundance observed in ^1^H NMR spectra.

**Figure 10:**
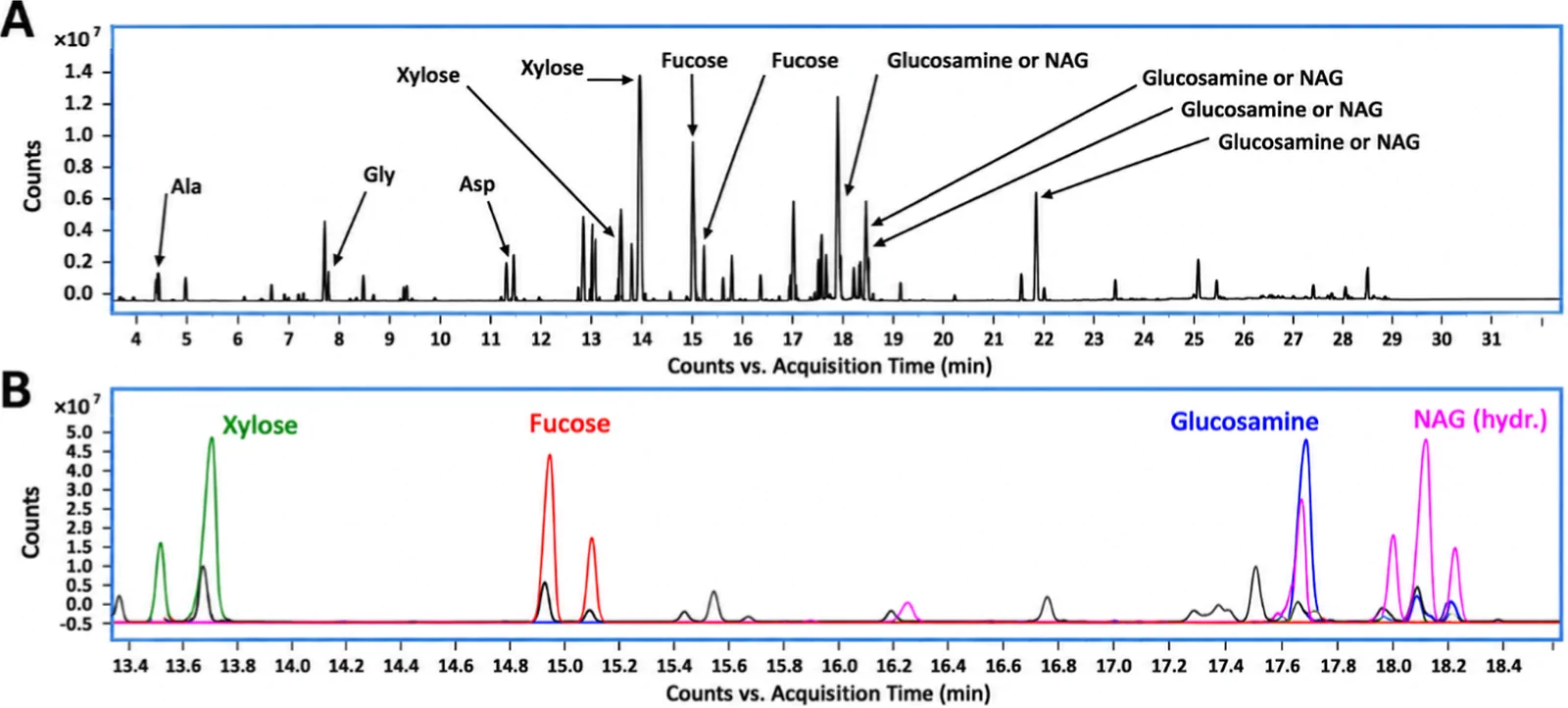
Gas chromatography–mass spectrometry (GC–MS) chromatograms of hydrolyzed and derivatized EPS and reference sugar standards. (A) EPS chromatogram showing amino acid assignments tentatively made using the National Institute of Standards and Technology (NIST) mass spectral library and sugar assignments supported by reference standards. (B) Overlay of the EPS chromatogram (black) and reference chromatograms of xylose (green), fucose (red), glucosamine (blue), and hydrolyzed *N*-acetylglucosamine (NAG; magenta), showing the retention-time region used for comparison.

The dominance of xylose in the EPS composition is noteworthy, as glucose is typically the major monosaccharide in cyanobacterial EPSs, and xylose-rich compositions are relatively rare.^6^ Xylose was identified as a major monosaccharide component of AQUAMEB-3 EPS. Xylose-containing polysaccharides have been associated with hydrophilic characteristics in previously reported microbial EPS systems.^80^

Fucose, a deoxyhexose sugar, introduces hydrophobic domains into the polymer structure, imparting amphiphilic characteristics. The coexistence of hydrophilic and hydrophobic moieties may be relevant to the interfacial properties of the EPS; however, the contributions of individual monosaccharide components to the observed emulsifying behavior were not independently evaluated in the present study. Moreover, fucose-containing EPSs have been linked to increased biological activity, including antioxidant and antibacterial effects.^6^

The detection of glucosamine and *N*-acetylglucosamine is particularly significant, as these amino sugars are rarely reported in EPSs of halotolerant cyanobacteria and are more commonly associated with alkaliphilic or freshwater species.^81,82^

Taken together, the compositional profile indicates that AQUAMEB-3 EPS is a heteropolysaccharide containing pentose (xylose), deoxyhexose (fucose), and amino sugar (glucosamine) units, together with ionizable functionalities. This compositional heterogeneity differs from the linear α-d-1,6-homoglucan reported for *Cyanothece* sp. 113.^47^

The coexistence of hydrophilic, hydrophobic, and ionizable functionalities may be associated with the physicochemical and functional behavior observed for AQUAMEB-3 EPS. However, because individual structural components were not fractionated or selectively evaluated, their respective contributions to the emulsifying, antioxidant, antibacterial, and metal-chelating activities cannot be independently established from the present data. These structure–property associations should therefore be interpreted as correlative rather than mechanistic.

Although cyanobacterial EPSs with physicochemical and biological activities have been previously reported, the present study extends this knowledge by providing an integrated characterization of an EPS produced by the halotolerant cyanobacterium *Euhalothece* sp. AQUAMEB-3.^83^ Previous studies have demonstrated the structural diversity and broad physicochemical and biotechnological potential of cyanobacterial EPSs.^43^ For example, Saad et al. (2024) characterized and purified an EPS from the halotolerant cyanobacterium *Acaryochloris* al-azhar MNE ON864448.1 and evaluated its antiviral activity, whereas Hazra et al. (2025) investigated EPS from marine cyanobacteria with emphasis on antioxidant activity and techno-functional properties, including water- and oil-holding capacities and flocculation.^84^ In the present study, detailed compositional and spectroscopic characterization was combined with molecular-weight analysis, surface charge, crystallinity, thermal behavior, morphology, rheology, emulsification, antioxidant and antibacterial activities, and cytocompatibility assessment within the same EPS system. Thus, the contribution of this study lies not in claiming universally superior performance, but in defining the distinct physicochemical and functional profile of an underexplored *Euhalothece*-derived EPS and expanding the available structure–property data set for cyanobacterial exopolysaccharides.

### Rheological Properties of EPS

The rheological behavior of *Euhalothece* sp. AQUAMEB-3 EPS solutions (0.1%, 0.5%, and 1.0% w/v) was evaluated by measuring apparent viscosity as a function of shear rate (Figure 11). The viscosity increased markedly with EPS concentration, reaching 22.02, 317.91, and 679.17 mPa·s at a shear rate of 10 s^–1^ for 0.1%, 0.5%, and 1.0% solutions, respectively. At higher shear rates (1000 s^–1^), viscosity values decreased substantially to 2.41, 5.99, and 10.09 mPa·s, confirming pronounced shear-thinning behavior.

**Figure 11:**
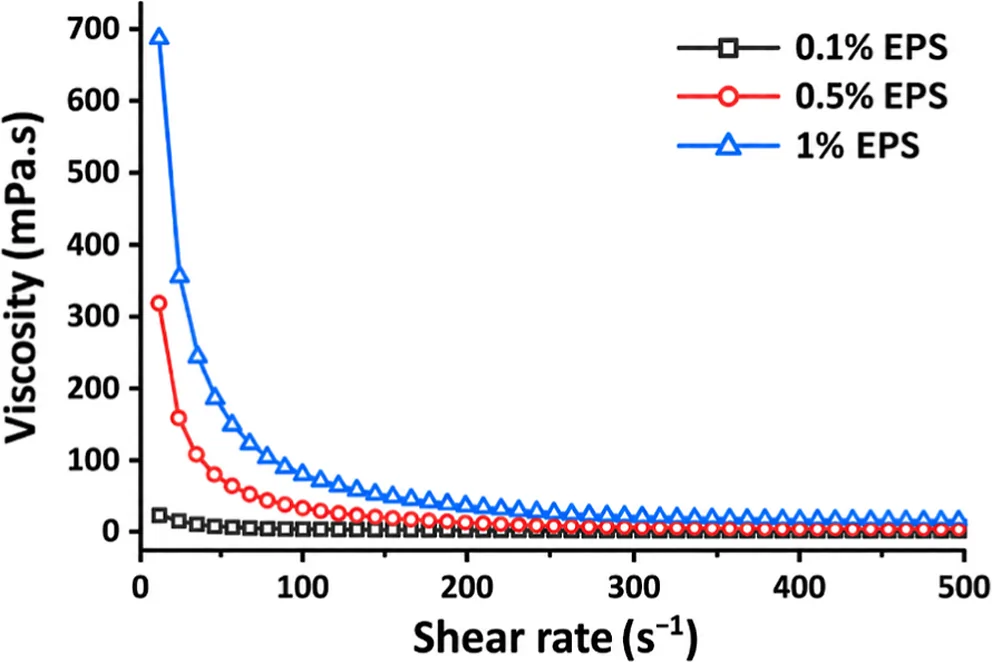
Viscosity as a function of shear rate in aqueous solutions of *Euhalothece* sp. AQUAMEB-3 exopolysaccharide (EPS) at concentrations of 0.1%, 0.5%, and 1.0% (w/v).

This non-Newtonian pseudoplastic behavior is characteristic of high-molecular-weight polysaccharides and is consistent with the elevated MW (2617 kDa), which promotes polymer chain entanglement and intermolecular interactions at low shear rates. When the shear rate is increased, the interactions between the chains are progressively disrupted, leading to alignment of polymer chains and a consequent reduction in flow resistance. Similar rheological profiles have been reported for other cyanobacterial EPS systems.^65,85^

Functionally, shear-thinning behavior provides significant advantages in practical applications. High viscosity at low shear rates ensures structural stability, suspension capability, and water retention, while reduced viscosity under shear facilitates flow, processing, and handling. This dual behavior is particularly beneficial for systems requiring both stability and processability.

In addition, the combination of pseudoplasticity and high molecular weight enhances interfacial stabilization and dispersion properties, supporting the emulsification performance of the EPS.^86^

### Emulsifying Properties of EPS

The emulsifying capacity of *Euhalothece* sp. AQUAMEB-3 EPS was evaluated using the emulsification index (*E*_24_) following 24 h interaction with liquid paraffin. All tested concentrations (0.1%, 0.5%, and 1.0% w/v) formed stable emulsions, with a clear concentration-dependent increase in emulsification efficiency (Figure 12), comparable to the xanthan gum used as a commercial reference.

**Figure 12:**
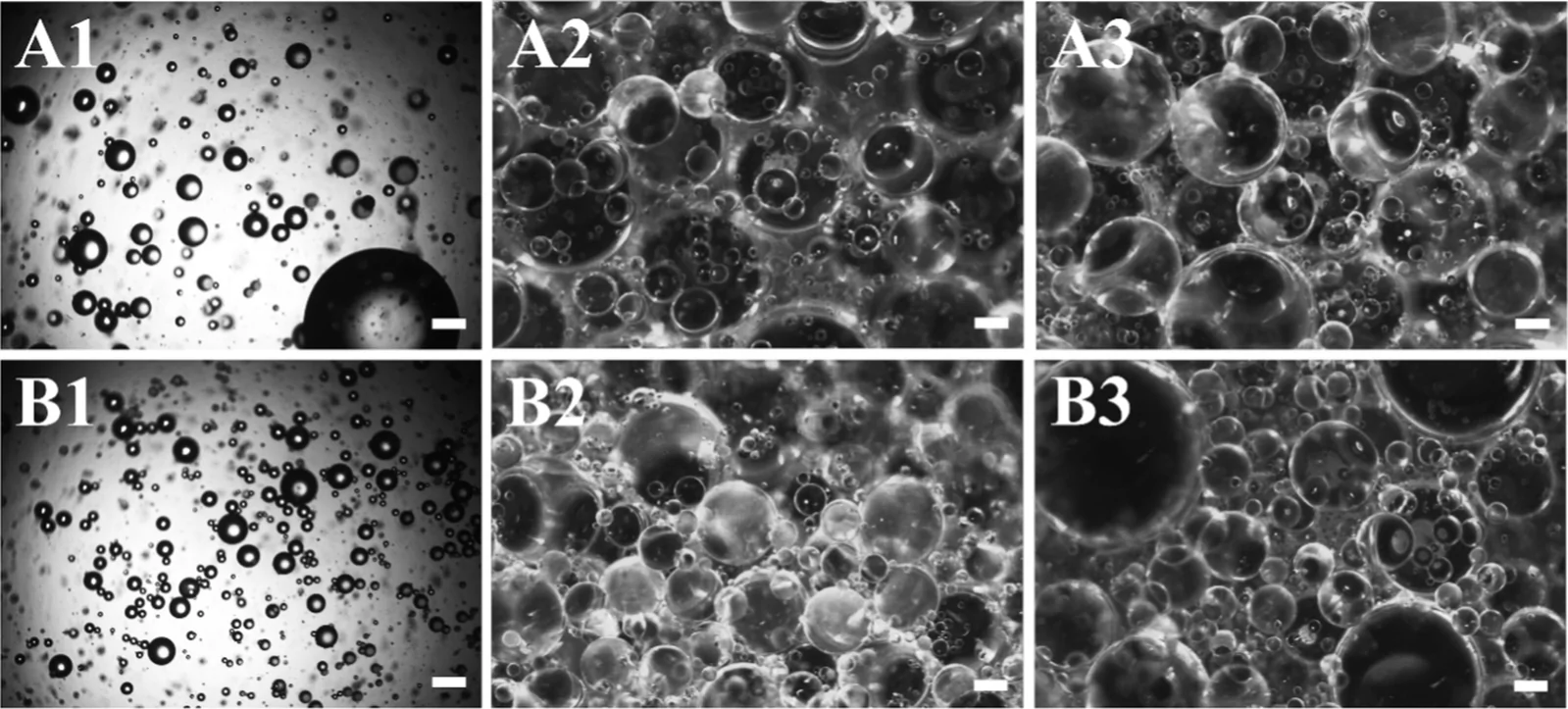
Optical microscope images of emulsion droplets: (A1,A2,A3); 1.0%, 0.5% and 0.1% (w/v) *Euhalothece* sp. AQUAMEB-3 exopolysaccharide (EPS)–liquid paraffin emulsions, respectively; (B1,B2,B3), 1.0%, 0.5% and 0.1% (w/v) xanthan gum–liquid paraffin emulsions, respectively. Scale bar = 400 μm.

*E*_24_ values (mean ± SD; *n* = 3) for EPS were 37.9 ± 4.3% (0.1%), 84.6 ± 4.5% (0.5%), and 94.2 ± 3.9% (1.0%), while xanthan gum exhibited values of 70.2 ± 10.8%, 95.3 ± 0.5%, and 100.0 ± 0.0% at the respective concentrations. Two-way ANOVA revealed significant effects of polymer (*F*_1_,_12_ = 41.91, *p* = 3.05 × 10^–5^) and concentration (*F*_2_,_12_ = 112.66, *p* = 1.67 × 10^–8^), as well as a significant interaction (*F*_2_,_12_ = 10.60, *p* = 0.00223), indicating that emulsification performance depends on both polymer type and concentration.

Post hoc analysis (Tukey HSD) demonstrated that EPS at 0.1% showed significantly lower emulsification compared to all other treatments, whereas at higher concentrations (0.5–1.0%), EPS exhibited comparable performance to xanthan gum, with overlapping statistical groups. These findings indicate that the difference between EPS and xanthan gum is most pronounced at low concentrations, while at higher concentrations the EPS approaches the performance ceiling of the assay. Specifically, EPS at 1.0% (w/v) was not significantly different from xanthan gum at the same concentration according to Tukey’s HSD test (Table 2).

**Table 2:** Emulsification Index after 24 h of Contact between Different Concentrations of Exopolysaccharide (EPS) or Xanthan Gum (0.1%, 0.5% and 1.0%, w/v) and Liquid Paraffin^a^

	0.1% (w/v)	0.5% (w/v)	1.0% (w/v)
*Euhalothece* sp. AQUAMEB-3 EPS	37.9 ± 4.3 **d**	84.6 ± 4.5 **ac**	94.2 ± 3.9 **ab**
xanthan gum	70.2 ± 10.8 **c**	95.3 ± 0.5 **ab**	100.0 ± 0.0 **b**

a Values are mean ± SD (sample SD; *n* = 3). Values sharing at least one lowercase letter are not significantly different, whereas values with no lowercase letter in common are significantly different among the six polymer × concentration groups (Tukey HSD, α = 0.05).

According to established criteria, emulsifiers capable of maintaining ≥50% emulsion stability after 24 h are considered effective.^87^ In this context, AQUAMEB-3 EPS at 0.5% and 1.0% clearly meets this threshold, confirming its strong emulsifying capability (Table 2). Moreover, the emulsions remained stable for at least one month (data not shown), highlighting the long-term interfacial stability provided by this EPS.

The high emulsification performance of AQUAMEB-3 EPS may be associated with its heterogeneous composition and the coexistence of hydrophilic, hydrophobic, and ionizable functionalities. Deoxy sugars such as fucose have been associated with hydrophobic interactions in microbial polysaccharides,^35^ whereas hydrophilic and anionic groups can promote hydration and electrostatic interactions at oil–water interfaces.^88^ These features have been reported to contribute to interfacial adsorption and emulsion stabilization in microbial EPS systems.^89^ However, the individual contributions of these structural features to the emulsifying behavior of AQUAMEB-3 EPS were not independently evaluated in the present study.

The high molecular weight and pseudoplastic rheological behavior of AQUAMEB-3 EPS may also be relevant to its emulsifying properties. High-molecular-weight microbial polysaccharides with shear-thinning behavior have been reported to support emulsion stability through rheological and interfacial effects.^90^ However, the specific contribution of these properties to the emulsification performance of AQUAMEB-3 EPS was not independently evaluated in the present study.

Compared with previously reported cyanobacterial EPSs, AQUAMEB-3 EPS exhibited relatively high emulsification indices. For example, cyanoflan from *Cyanothece* sp. CCY 0110 has been reported to exhibit *E*_24_ values of 51–64%, whereas AQUAMEB-3 EPS reached 84.6% and 94.2% at 0.5% and 1.0% (w/v), respectively.^35^

Overall, the observed emulsifying behavior of AQUAMEB-3 EPS may be associated with its molecular weight, charge, and heterogeneous composition; however, the individual contributions of these structural features cannot be resolved from the present data.

### Antibacterial Activity Tests of EPS

The antibacterial activity of *Euhalothece* sp. AQUAMEB-3 EPS was evaluated against *E. coli* (ATCC 25922) and *S. aureus* (ATCC 25923) using the broth microdilution method over a concentration range of 39–2500 μg mL^–1^. The EPS exhibited clear antibacterial activity against both Gram-negative and Gram-positive bacteria, with minimum inhibitory concentration (MIC) values of 150 μg mL^–1^ for *E. coli* and 300 μg mL^–1^ for *S. aureus*.

Interestingly, the EPS showed higher activity against Gram-negative *E. coli* compared to Gram-positive *S. aureus*, which contrasts with the general trend reported for many cyanobacterial EPSs. Reported MIC values for EPSs produced from *Gloeocapsa*, *Synechocystis*, *Nostoc* spp., and *Rhodella reticulata* range between 125 and 1000 μg mL^–1^ against *S. aureus*,^91,92^ while significantly higher concentrations (2500–10,000 μg mL^–1^) have been reported for activity against both *E. coli* and *S. aureus* in other systems.^2^ In this context, AQUAMEB-3 EPS demonstrates comparable or improved antibacterial activity.

The antibacterial activity of AQUAMEB-3 EPS may be associated with its physicochemical properties, although the underlying mechanism was not directly investigated. Considering its high molecular weight (2617 kDa), AQUAMEB-3 EPS is unlikely to penetrate the bacterial cell wall and directly disrupt the cytoplasmic membrane. Instead, it is more plausible that the EPS interacts with accessible components of the bacterial cell envelope, including the peptidoglycan layer and, in Gram-negative bacteria, the outer membrane, through electrostatic interactions. Such adsorption may lead to the formation of a hydrated polymeric layer on the cell surface, which could interfere with nutrient transport, ion exchange, and other cell-envelope-associated processes. These mechanisms remain hypothetical and require confirmation through membrane-permeability, leakage, ultrastructural, and bacterial surface-charge analyses.^93^

### Antioxidant and Ferrous Ions Chelating Activity of EPS

The antioxidant activity of AQUAMEB-3 EPS was evaluated using the DPPH radical scavenging assay. The EPS exhibited concentration-dependent free radical scavenging activity, reaching a maximum inhibition of 82.6 ± 25.8% at 8.8 mg mL^–1^ (Figure 13A). The relatively large standard deviation observed at the highest EPS concentration (8.8 mg mL^–1^) is likely attributable to partial aggregation/precipitation of the polymer in the methanolic reaction medium. The resulting turbidity and sample heterogeneity may interfere with spectrophotometric absorbance measurements, thereby increasing the variability of the measured DPPH scavenging activity. Therefore, the antioxidant activity at this concentration should be interpreted with appropriate caution. The half-maximal effective concentration (EC_50_) was theoretically estimated by linear interpolation between adjacent data points surrounding 50% inhibition, yielding an approximate value of 4.05 mg mL^–1^. One-way ANOVA revealed a statistically significant effect of concentration on radical scavenging activity (*p* < 0.01). Post hoc analysis (Tukey HSD) indicated that higher concentrations (4.4 and 8.8 mg mL^–1^) exhibited significantly greater inhibition compared to lower concentrations (0.3–1.1 mg mL^–1^). Ascorbic acid, included as a positive reference control, exhibited approximately 100% DPPH radical-scavenging activity across the tested concentration range, providing a reference benchmark for the EPS response.

**Figure 13:**
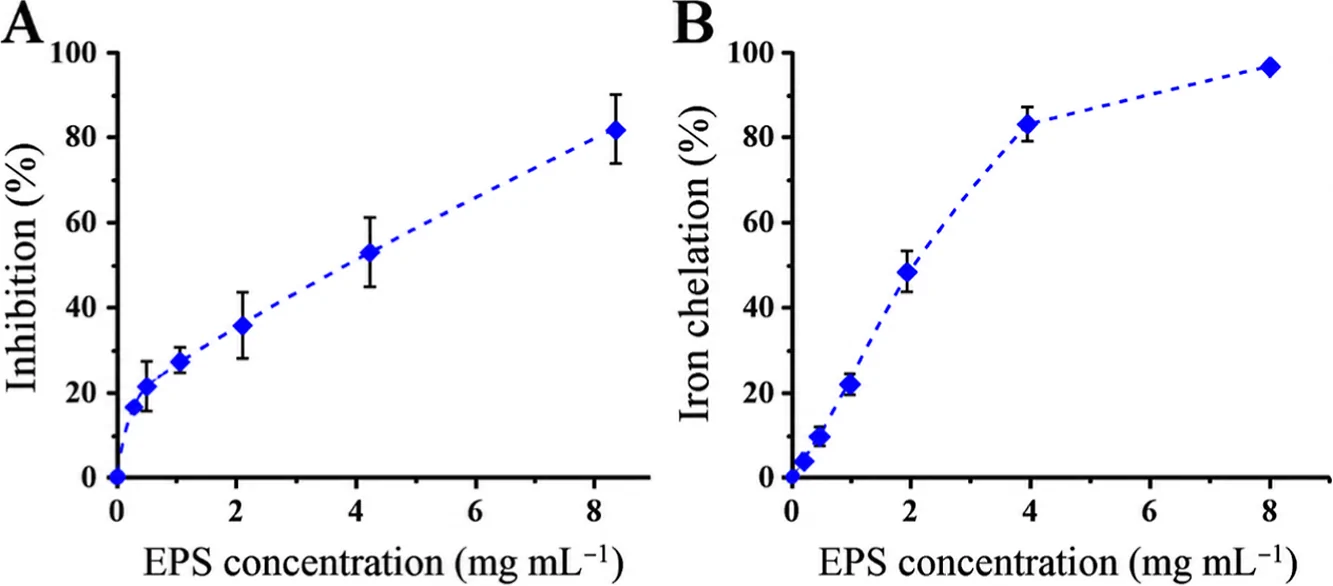
(A) Scavenging properties of the *Euhalothece* sp. AQUAMEB-3 EPS in a concentration range of 0.3–9 mg mL^–1^ assayed against 2,2-diphenyl-1-picrylhydrazyl (DPPH) radical. Ascorbic acid was used as a positive reference control and exhibited approximately 100% radical-scavenging activity across the tested concentration range. (B) Fe^2+^-chelating activity of the isolated EPS over a concentration range of 0.25–8 mg mL^–1^, determined using the ferrozine assay.

Polysaccharides such as AQUAMEB-3 EPS can act as hydrogen or electron donors, neutralizing free radicals and terminating chain reactions. This activity is closely linked to the presence of functional groups such as hydroxyl (−OH) and carboxyl (−COOH) moieties, particularly those associated with uronic acids.^92^

In addition to radical scavenging, the EPS demonstrated strong ferrous ion (Fe^2+^) chelating activity. The chelation **activity** increased with concentration, reaching approximately 96% at 8 mg mL^–1^ (Figure 13B), while only ∼4% chelation was observed at 0.25 mg mL^–1^. This concentration-dependent response indicates substantial Fe^2+^-chelating activity under the tested conditions. One-way ANOVA revealed a statistically significant effect of concentration on chelating activity (*p* < 0.001). Post hoc analysis (Tukey HSD) indicated that higher concentrations (4 and 8 mg mL^–1^) exhibited significantly greater chelation compared to lower concentrations (0.25–1 mg mL^–1^), while 2 mg mL^–1^ represented a transition range. No significant difference was observed between 4 and 8 mg mL^–1^, indicating a plateau in the measured chelating activity within the tested concentration range. The half-maximal effective concentration (EC_50_) was theoretically estimated by linear interpolation, yielding an approximate value of 2.1 mg mL^–1^. The lower EC_50_ value observed for Fe^2+^ chelation compared with DPPH scavenging indicates that a lower EPS concentration was required to reach 50% activity in the chelation assay under the tested conditions. However, the present assay provides a measure of relative Fe^2+^-chelating activity rather than quantitative metal-binding capacity or binding stoichiometry. Further studies determining metal-binding capacity and EPS–metal stoichiometry would be required to quantitatively characterize the metal-binding behavior of AQUAMEB-3 EPS and enable direct comparison with other cyanobacterial EPS systems.

The observed antioxidant and chelating activities are consistent with literature reports for cyanobacterial EPSs, which typically exhibit 20–60% radical scavenging and 30–85% iron chelation at higher concentrations (∼20 mg mL^–1^).^39^ Notably, AQUAMEB-3 EPS achieved comparable or activity at lower concentrations, suggesting relatively high efficiency. For example, EPS from *N. carneum* exhibited ∼15% scavenging at 5 mg mL^–1^,^65^ whereas AQUAMEB-3 EPS reached approximately 60% under similar conditions. Similarly, reported IC_50_ values for EPS from *Leptolyngbya* sp. were 4 mg mL^–1^ (DPPH) and 40 mg mL^–1^ (iron chelation),^94^ indicating that AQUAMEB-3 EPS achieves effective activity within comparable or lower concentration ranges.

Taken together, the observed antioxidant and antibacterial properties of AQUAMEB-3 EPS may be associated with its physicochemical and compositional characteristics. Uronic acids and other ionizable groups may contribute to the observed metal-chelating and radical-scavenging behavior, while other compositional and macromolecular features may also influence its biological interactions. However, these associations should be interpreted as correlative, since the contributions of individual structural components were not independently evaluated in the present study.^93^

Such a combination of properties is particularly advantageous for advanced applications where both stabilization and biological activity are required. For instance, materials with combined antioxidant and antibacterial properties are highly desirable for biomedical formulations and protective systems, where control of oxidative stress and microbial contamination is critical.^94^

### Cytotoxicity Assessment of EPS

The cytocompatibility of *Euhalothece* sp. AQUAMEB-3 EPS was evaluated using the MTT assay on BEAS-2B, A549, and L929 cell lines over a concentration range of 5–2000 μg mL^–1^ (Figure 14), where BEAS-2B (normal human bronchial epithelial cells) were used to represent noncancerous epithelial cells, A549 (human lung carcinoma cells) to assess potential selective anticancer effects, and L929 (mouse fibroblast cells) as a standard model for cytotoxicity testing in accordance with ISO guidelines (Figure 14). The results revealed a clear concentration-dependent response, with high cell viability observed at low to moderate EPS concentrations and reduced viability at higher concentrations.

**Figure 14:**
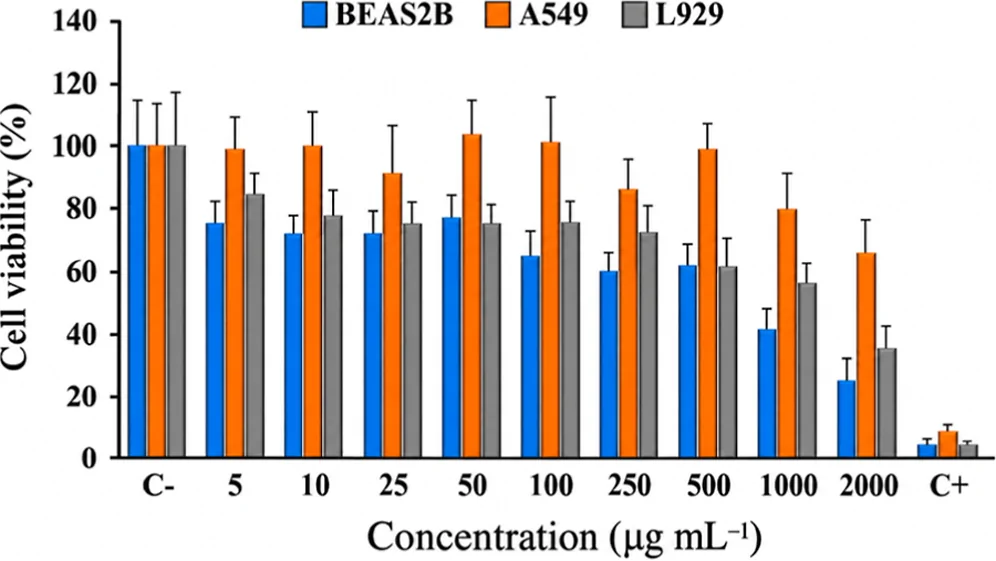
Cell viability (%) of BEAS-2B human bronchial epithelial cells, A549 human lung carcinoma cells, and L929 mouse fibroblasts cell lines following exposure to varying concentrations (5–2000 ppm) of the tested sample, as determined by the 3-(4,5-dimethylthiazol-2-yl)-2,5-diphenyltetrazolium bromide (MTT) assay. Cells treated with culture medium alone (C^–^) served as the negative control (100% viability), while cells treated with medium containing 1% Triton X-100 (C^+^) served as the positive control. Data are presented as mean ± standard deviation (*n* = 3). A concentration-dependent decrease in cell viability was observed, with differential sensitivity among the cell lines.

At lower concentrations (5–100 μg mL^–1^), the EPS exhibited excellent cytocompatibility across all tested cell lines, with cell viability generally exceeding 80–100%. A549 cells maintained near-complete viability (∼100%) within this range, while BEAS-2B and L929 cells showed slightly lower but still acceptable viability levels (∼70–85%), indicating minimal cytotoxic effects.

At intermediate concentrations (250–500 μg mL^–1^), a gradual decrease in viability was observed, particularly for BEAS-2B and L929 cells. In contrast, A549 cells remained highly tolerant, maintaining viability values close to or above 90%, suggesting a cell type-dependent response to EPS exposure.

At higher concentrations (≥1000 μg mL^–1^), a more pronounced cytotoxic effect became evident. BEAS-2B cells exhibited significant sensitivity, with viability decreasing to approximately 40% at 1000 μg mL^–1^ and 25% at 2000 μg mL^–1^. L929 cells showed moderate sensitivity (∼55% and ∼35%, respectively), whereas A549 cells remained comparatively resistant, retaining ∼80% viability at 1000 μg mL^–1^ and ∼65% at 2000 μg mL^–1^.

According to ISO 10993-5 criteria, materials that maintain cell viability above 70% are considered noncytotoxic (ISO 10993-5:2009). Based on this threshold, AQUAMEB-3 EPS can be classified as noncytotoxic at concentrations up to approximately 500–1000 μg mL^–1^, defining a favorable biocompatibility window for potential applications.

The observed cytotoxicity at higher concentrations may be attributed to the physicochemical characteristics of the EPS. Its high molecular weight (2617 kDa) and strong anionic charge (zeta potential −45.1 mV) may influence cell–polymer interactions, increase medium viscosity, and limit mass transport, thereby affecting cellular metabolism at elevated concentrations. In addition, the dense polymer network may alter diffusion of nutrients and oxygen in vitro, contributing to reduced cell viability.^95^

The higher tolerance observed in A549 cells compared to BEAS-2B and L929 cells is likely associated with their cancerous origin, as tumor-derived cell lines are generally more resistant to environmental stress and external agents.

Overall, these findings demonstrate that AQUAMEB-3 EPS possesses a favorable safety profile within a defined concentration range, supporting its potential use in biomedical applications. Nevertheless, further investigations, including long-term cytocompatibility, proliferation assays, and in vivo evaluations, are required to fully establish its biocompatibility and translational potential.

## Conclusion

The EPS produced by *Euhalothece* sp. AQUAMEB-3 was identified as a structurally complex anionic heteropolysaccharide composed of neutral sugars (notably xylose and fucose), uronic acids, and minor protein fractions. The EPS also exhibited a high weight-average molecular weight (*M*_w_ = 2617.3 kDa) and a strong negative surface charge (−45.1 mV), further defining its physicochemical profile.

The EPS exhibited pronounced shear-thinning behavior, high emulsification capacity, and thermal stability. Its emulsification index (*E*_24_) reached 94.2% at 1.0% (w/v), demonstrating performance comparable to xanthan gum under the tested conditions. Importantly, the EPS demonstrated significant bioactivity, including antibacterial effects against both Gram-positive and Gram-negative bacteria, as well as notable antioxidant and Fe^2+^-chelating capacities.

The integration of bioactivity with favorable physicochemical properties provides a distinctive functional profile for AQUAMEB-3 EPS. These findings suggest that AQUAMEB-3 EPS represents a promising candidate for further investigation as a structural and functional biopolymer.

Overall, the present study provides a comprehensive physicochemical and functional characterization of EPS produced by *Euhalothece* sp. AQUAMEB-3 and defines its compositional, physicochemical, and functional profile. These findings support further investigation of this EPS for potential biotechnological applications. Future studies focusing on process optimization, purification, targeted structural modification, and application-specific evaluation will be necessary to establish causal structure–function relationships and validate its practical use.

## Data Availability

Data is available upon request.
